# Diet effects on colonic health influence the efficacy of Bin1 mAb immunotherapy for ulcerative colitis

**DOI:** 10.1038/s41598-023-38830-2

**Published:** 2023-07-21

**Authors:** Sunil Thomas, Nickey Dilbarov, Joseph Kelly, Giancarlo Mercogliano, George C. Prendergast

**Affiliations:** 1 Lankenau Institute for Medical Research, 100 E. Lancaster Avenue, Wynnewood, PA 19096 USA; 2 Lankenau Medical Center, Wynnewood, PA 19096 USA

**Keywords:** Cell biology, Immunology

## Abstract

Ulcerative colitis (UC) is an idiopathic disease of the large intestine linked to high fat-high protein diets, a dysbiotic microbiome, and a metabolome linked to diet and/or aberrant circadian rhythms associated with poor sleeping patterns. Understanding diet-affected factors that negatively influence colonic health may offer new insights into how to prevent UC and enhance the efficacy of UC immunotherapy. In this preclinical study, we found that standard or high fiber diets in mice positively influenced their colonic health, whereas a high fat-high protein diet negatively influenced colonic health, consistent with clinical findings. Animals fed a high fat/high protein diet experienced obesity and a reduced colon length, illustrating a phenotype we suggest calling *peinosis* [hunger-like-condition; Greek, *peina*: hunger; *osis*: condition], as marked by a lack of nutrient energy remaining in fecal pellets. Notably, a high fat/high protein diet also led to signs of muscle weakness that could not be explained fully by weight gain. In contrast, mice on a high fiber diet ranked highest compared to other diets in terms of colon length and lack of muscle weakness. That said, mice on a high fiber diet were more prone to UC and toxic responses to immunotherapy, consistent with clinical observations. Recent studies have suggested that a standard diet may be needed to support the efficacy of immunotherapeutic drugs used to prevent and treat UC. Here we observed that protection against UC by Bin1 mAb, a passive UC immunotherapy that acts by coordinately enforcing intestinal barrier function, protecting enteric neurons, and normalizing the microbiome, was associated with increased colonic levels of healthful short-chain fatty acids (SCFA), particularly butyric acid and propionic acid, which help enforce intestinal barrier function. This work offers a preclinical platform to investigate how diet affects UC immunotherapy and the potential of dietary SCFA supplements to enhance it. Further, it suggests that the beneficial effects of passive immunotherapy by Bin1 mAb in UC treatment may be mediated to some extent by promoting increased levels of healthful SCFA.

## Introduction

The major inflammatory bowel diseases (IBD) include Crohn’s disease and Ulcerative colitis (UC). Crohn’s disease is characterized by inflammation and ulceration of both the small and large intestines, whereas UC is characterized by ulceration of the large intestine (colon). UC is an idiopathic inflammatory disease of the colon that starts in the rectum and extends proximally. While the basis for its patterns of etiology, pathophysiology and progression remain poorly understood^1–5^, a variety of environmental factors contribute to UC development, including altered circadian rhythms^6,7^, high fat/high protein diets^8,9^, alcohol abuse^10,11^, and dysbiotic microbiomes plus their associated metabolomes^12^. In UC patients, smoking further increases the risk of cancer and mortality in UC patients^13^. Gaining deeper understanding of the dietary factors that influence and enforce colonic health is important to learn how to reduce UC risk and flares. While immunotherapies to treat UC and prevent UC flares continue to be developed, recent studies suggest that their efficacy may be influenced by dietary factors. Accordingly, there is a need for models to investigate and identify these factors, with the goal of learning how to maximize the efficacy of such immunotherapeutic agents.

We have investigated the Bin1 monoclonal antibody 99D (Bin1 mAb) as a highly active passive immunotherapy to prevent chemically-induced UC in mouse models^1–3^. Bin1 is one of two BAR adapter encoding genes conserved throughout eukaryotic evolution, expressed in complex patterns of alternately spliced cytoplasmic or nucleocytoplasmic splice isoforms^14,15^. Bin1 proteins have been implicated in diverse cellular processes, including polarized cell stress responses, endocytosis, programmed cell death, actin organization, and transcriptional control. The *BIN1* gene includes 20 exons that encode an N-terminal BAR domain, a phosphoinositide (PI) binding motif, a CLAP (clathrin and AP2) binding domain, a Myc-binding domain (MBD) and an Src homology 3 (SH3) domain. The N-terminal BAR domain in Bin1 has a canonical function in controlling membrane curvature and a moonlighting function in controlling transcriptional activity^15^. Preclinical genetic and pharmacological studies have demonstrated that Bin1 attenuation abolishes sensitivity to experimental chemically-induced colitis in association with an enhancement of intestinal epithelial cell barrier function^1,2,16^. Specifically, we previously demonstrated that mice treated with the cell-penetrating Bin1 mAb protects against susceptibility to chemically-induced colitis, acting through several mechanisms to pleiotropically block colonic inflammation^1–3^. Recent reports suggest that diet can strongly affect the efficacy and quality of immunotherapeutic treatments for UC^17,18^. Hence, in this study we investigated (a) how diet influences the colon physiology and (b) how diet affects Bin1 mAb 99D activity as a highly active passive immunotherapy to prevent and treat UC.

## Materials and methods

### Mouse UC model

The established dextran sodium sulfate (DSS)-induced model of ulcerative colitis was used as described previously^1–3^. Animals were weighed before, during and after treatments. Both short term (30 days) and long-term dietary studies (7 months) were performed. All experimental protocols were approved by the institutional animal care and use committee (IACUC: A14-3006) of Lankenau Institute for Medical Research (LIMR) and were conducted in accordance with the guidelines of the National Institutes of Health (NIH). All methods were carried out in accordance with relevant guidelines and regulations.

For short term study (30 days), C57BL/6 male mice (Jackson Laboratory, USA) of 5 weeks of age were fed with the indicated diets. Studies in animal model of colitis are performed in five-week-old mice. The diet is switched from standard diet to other diets (fiber, carbohydrate, high fat/high protein) on week five after its arrival. They are fed with the new diet for one month so that the physiology and microbiome respond to the new diet.

On day 25, animals were fed 3% dextran sodium sulfate (DSS, Alfa Aesar, MW 40 kDa) dissolved in drinking water ad libitum (n = 5 per group). After 6 days, mice were provided distilled water. Starting 24 h after feeding distilled water, mice were injected i.p. (0.5 mg of purified antibody per mouse) with Bin1 mAb 99D or antibody isotype control as described^1^. Seven days later, mice were euthanized by CO_2_ inhalation and colons were dissected and inspected for gross macroscopic lesions.

For long term study (7 months), to determine the response of diet with aging, C57BL/6 male mice of 5 weeks of age were fed with the indicated diets. On day 1 of the seventh month, animals were fed 3% DSS in drinking water ad libitum and then treated as noted above in the short-term study (n = 5 per group). Mice fed a high fiber diet expired 4 days after i.p. administration of Bin1 mAb 99D. Hence, mice on a high fiber diet were fed drinking water for 7 days after DSS treatment, followed by injection with the Bin1 mAb 99D, which enabled the survival of all animals. All methods in animals were performed in accordance with ARRIVE guidelines (Fig. S1).

### Animal diets

The following diets were fed to animals (TestDiet/LabDiet, Divison of Land O’Lakes Purina Feed, USA):

*Standard diet*: Protein 19%, Fat 11%, Fiber 2.4%, Carbohydrate 52%, supplemented with essential vitamins and minerals.

*Standard diet* + *antibiotic*: Same as above with Amoxicillin (0.25 mg/ml of drinking water). The antibiotic was provided ad libitum in drinking water.

High carbohydrate diet: Protein 10%, Fat 2.1%, Fiber 4.9%, Carbohydrate 78.5%, supplemented with essential vitamins and minerals.

*High fiber diet*: Protein 10%, Fat 4.1%, Fiber 19.6%, Carbohydrate 64.4%, supplemented with essential vitamins and minerals.

*High fat/high protein diet*: Protein 30%, Fat 28.5%, Fiber 0%, Carbohydrate 32.9%, supplemented with essential vitamins and minerals.

The ingredients and nutritional profile of the diets are provided in supplementary Tables 1–4. In this study we did not determine the food consumed by each animal group every week. We filled the food rack of all the cages at the start of the week.

### Motor performance assessments

The rotarod has been shown to be used for the study of animal models of muscle weakness and peripheral neuropathies^19^. Motor performance of the animals was assessed using the rotarod (Ugo Basile, Italy) with slight modifications to the procedure of Varghese et al.^20^ Briefly, mice were placed on the rotarod, which was set to accelerate from 4 to 40 rotations per minute over a period of 200 s. The time at which the mice could no longer hold onto the rotarod was recorded as latency to fall. Each mouse was tested two times per day for the duration of the study. The test was conducted in a room with minimal disturbances of sound, movement, light, and temperature. A hanging wire test (wire hang test) was conducted by placing the animals on the top of a wire mesh lid. The wire mesh was slowly turned upside down and the latency of animals to fall off the wire mesh was measured in seconds (up to a maximum of 120 s).

### Immunohistochemistry

Colonic tissues dissected from euthanized mice were washed in PBS and were processed for immunohistochemistry as described^1^. Fixed and mounted tissues were probed with antibodies for neuronal nuclear protein (NeuN), and glial fibrillary acidic protein (GFAP) (Cell Signaling, MA), according to the manufacturer’s instructions. Cells were mounted with Fluoromount-G (Southern Biotech, AL) and visualized by confocal microscopy (Nikon Eclipse TI, Japan). Images were taken from different fields from the same slide. The experiments were repeated three times. The corrected total cell fluorescence (CTCF) was analyzed using Image J, the open-source image analysis software.

### Western blot analysis

Bin1 mAb treated or control colons were dissected and lysed as previously described^1,2^. After polyacrylamide gel electrophoresis (10% gel) and transfer to nitrocellulose membrane, they were probed with antibodies for claudin proteins (Claudin‐5, -7), BMI-1, Fas and Actin; 1–2 μg mL − 1; Life Technologies, USA; Cell Signaling Technology, USA) according to the manufacturer’s instructions. The protein band density was quantitated using Image J.

### Gut microbiome analyses

Mice were placed on a raised platform to determine the quantity of fecal pellets produced in 10 minutes^3^. We collected 4–8 fecal pellets in 10 min from untreated control mice; hence, we monitored mice for 10 min to compare production of fecal pellets in each experimental cohort. Placing the animals on a raised platform improved fecal pellet collection compared to leaving the animals in a container for the same duration. The microbiome of the fecal pellet was analyzed directly by extracting microbial DNA with the DNeasy PowerSoil Kit (QIAGEN), following directions of the manufacturer. Samples were subjected to 16S rRNA sequencing for taxonomic identification (Arizona State University Microbiome Core). Experiments were repeated three times.

### Microbiome library preparation methodology

Bacterial community analysis was performed via next generation sequencing in MiSeq Illumina platform, as described in detail in Thomas et al.^3^

### Microbiome downstream analysis

Downstream visualization and statistics were performed by the Harvard T.H. Chan School of Public Health Microbiome Analysis Core, as described in detail in Thomas et al.^3^

### Metabolome analysis

Fecal pellets from mice on different diets were cultured anaerobically in a biosimulator in LB medium for 3 days, as described in Thomas^21^. After centrifugation at 8000 g for 10 min, short chain fatty acids (SCFAs) were quantified using a Waters Acquity UPLC System with a Photodiode Array Detector and a HSS T3 1.8 μm 2.1 × 150 mm column. The flow rate was set to 0.25 mL/min, the injection volume was 5 uL, the column temperature was 40 °C, the sample temperature was 4 °C, and the run time was 25 min per sample. Eluent A was 10 mM sodium phosphate monobasic, pH 2.5; eluent B was methanol; 0.1% formic acid in water was used for the weak needle wash; the strong needle wash was 0.1% formic acid in acetonitrile; the seal wash was 10% acetonitrile in water. The gradient was 100% eluent A for 5 min, followed by 70% eluent B from 5 to 22 min, and then 100% eluent A for 3 min. The photodiode array was set to read absorbance at 215 nm with 4.8 nm resolution. Samples were quantified against standard curves of at least five points run in triplicate. Standard curves were run at the beginning and end of each metabolomics run. Quality control checks (blanks and standards) were run at every eight samples^22^.

### Statistical analysis

Results are expressed as mean ± SD. Unpaired two‐tailed Student t-tests and one-way ANOVA was used to compare sets of data obtained from independent groups. Statistical significance was considered at the level of *P* < 0.05. Statistical analysis was performed with GraphPad Prism 8.0 (GraphPad Software, CA, USA).

## Results

### Diet influences colon length

**Figure 1:**
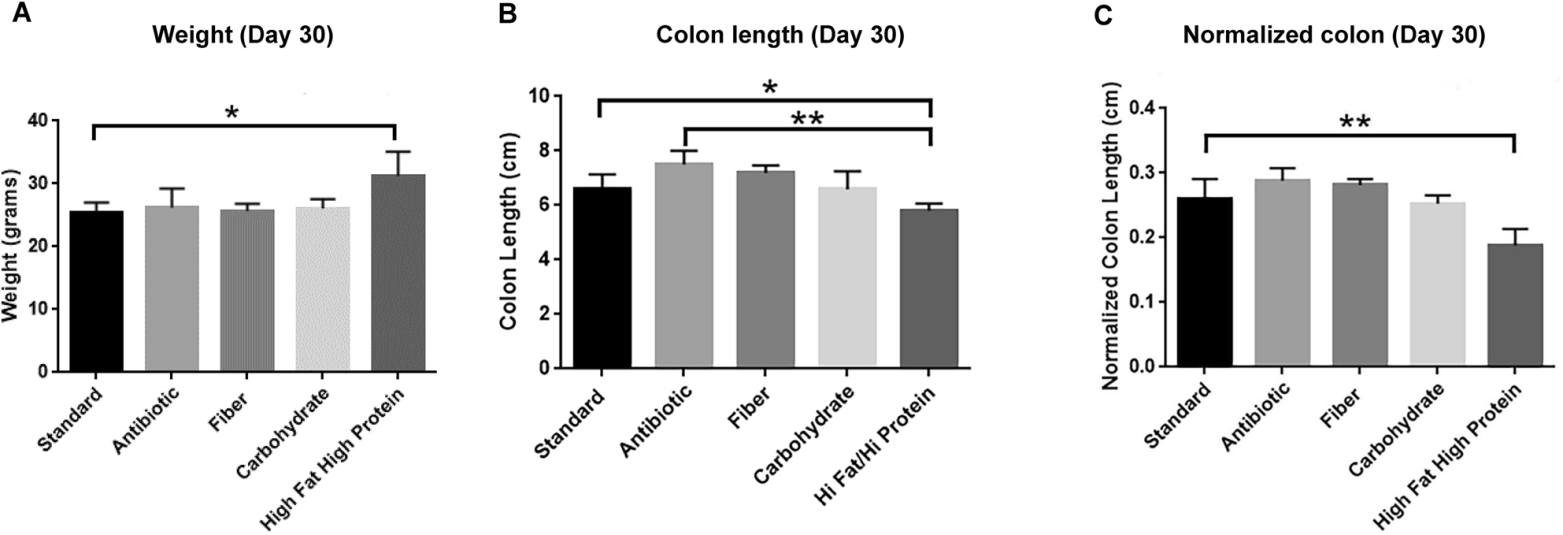
Phenotypic effects of diet on mice. There was a change in (**A**) weight of mice, (**B**) length of colon, (**C**) normalized colon, after feeding with a high fat/high protein diet for 30 days (*P < 0.05, **P < 0.01, as determined by t test). All results are expressed as Mean ± SD; (n = 5 mice per treatment).

In a short-term study, we determined the weight of mice after feeding with different diets for 30 days. The animals fed with a high fat/high protein diet gained weight compared to mice fed with the standard diet (*P* < 0.05) (Fig. 1A). The length of the colon of the mice were measured after 30 days on different diets. Feed containing antibiotic produced the longest colon In contrast, a high fat/high protein diet produced a significant reduction in colon length compared to mice fed with a standard diet (*P* < 0.05) (Fig. 1B). Normalization of the body weight with colon length also demonstrated significant shorter colon in mice fed with a high fat/high protein diet compared to mice fed with standard diet (*P* < 0.01) (Fig. 1C). We concluded that a high fat/high protein diet was associated with a decrease in colon length, suggesting a distinct gut physiology associated with this diet.

### Diet influences the status of enteric neurons

**Figure 2:**
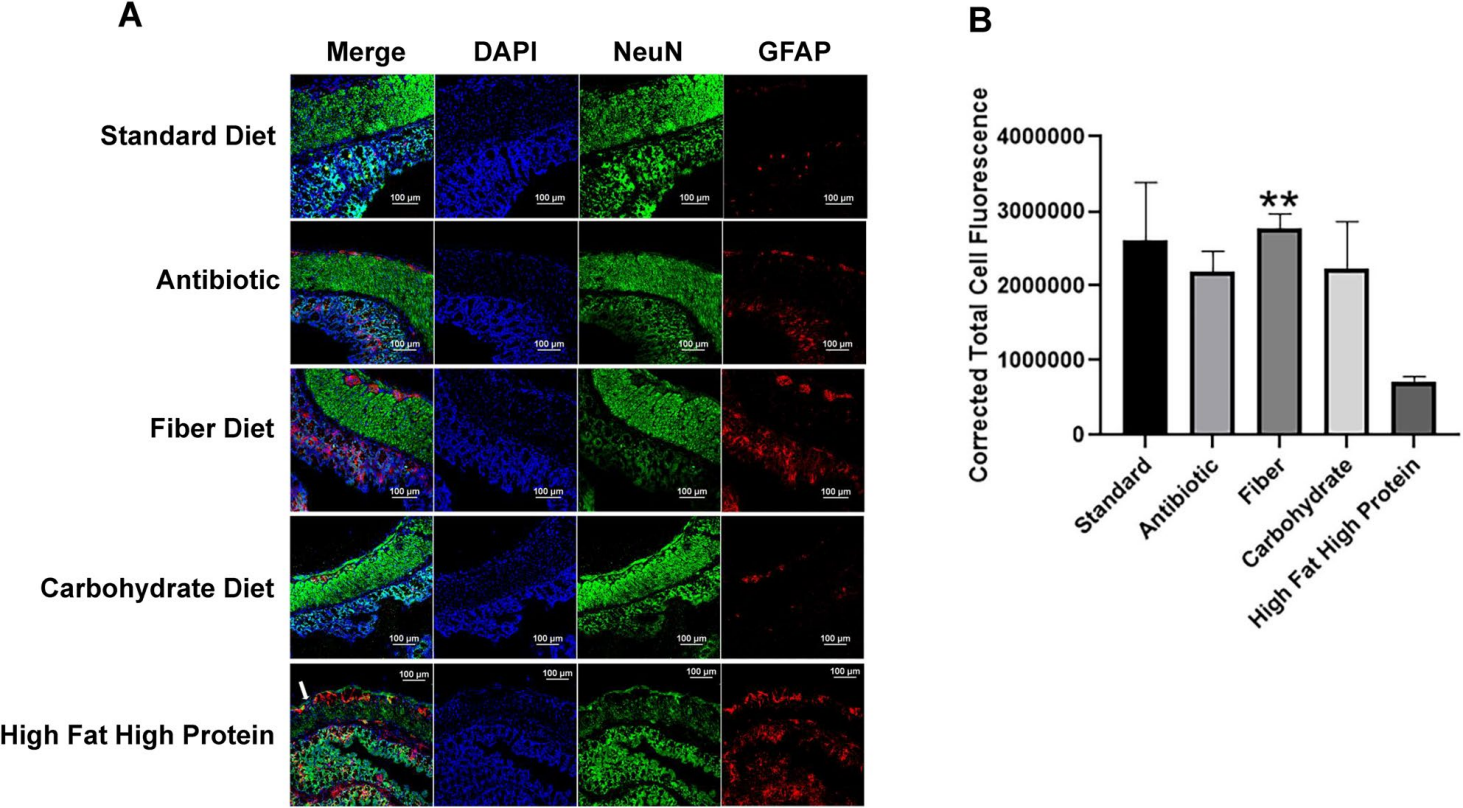
High fat/high protein diet decreased NeuN expressing enteric neurons in the colon muscularis mucosa. (**A**) The muscularis mucosa of the colon of mice treated with standard, antibiotic, fiber and carbohydrate diets had good NeuN expressing enteric neurons compared to colon from mice fed with a high fat/high protein diet. However, the mucosal layer of the colon from high fat/high protein diet had high expression of the NeuN enteric neurons and GFAP. The figures are representative of three images. (**B**) The colon of mice on a high fat high protein diet had low levels of NeuN expression in the muscularis mucosa as quantitated by CTCF analysis (CTCF analysis, n = 3 mice tissues per treatment) (**P < 0.01, as determined by one-way ANOVA).

The enteric nervous system (ENS) is an autonomous nervous system of the gastrointestinal (GI) tract that orchestrates GI behaviors independently of the central nervous system (CNS). ENS dysfunction is associated with digestive disorders^3^. We recently demonstrated that enteric neurons are altered during UC and the alterations are reversed by treatment with Bin1 mAb passive immunotherapy^3^. Since it is not known whether diets per se influence enteric neurons, we evaluated the number and distribution of the ENS system in mice fed different diets by NeuN and GFAP immunostaining of gut tissue neurons. NeuN expression was robust in the gut muscularis mucosa on all diets, except for the high fat/high protein diet where NeuN expression was markedly lower (Fig. 2A). The CTCF data confirmed low expression of NeuN in the muscularis mucosa of the colon from mice fed on a high fat high protein diet compared to other diets (Fig. 2B) (**P < 0.01, as determined by one-way ANOVA). In contrast, GFAP expression was relatively higher in the mucosal layer of mice fed a high fat/high protein diet, compared to the level on other diets. These observations suggested that a high fat/high protein diet may affect the physiological status of enteric neurons.

### Differential effects of diet on tight junction and stem cell response to Bin1 mAb immunotherapy in DSS-induced UC

**Figure 3:**
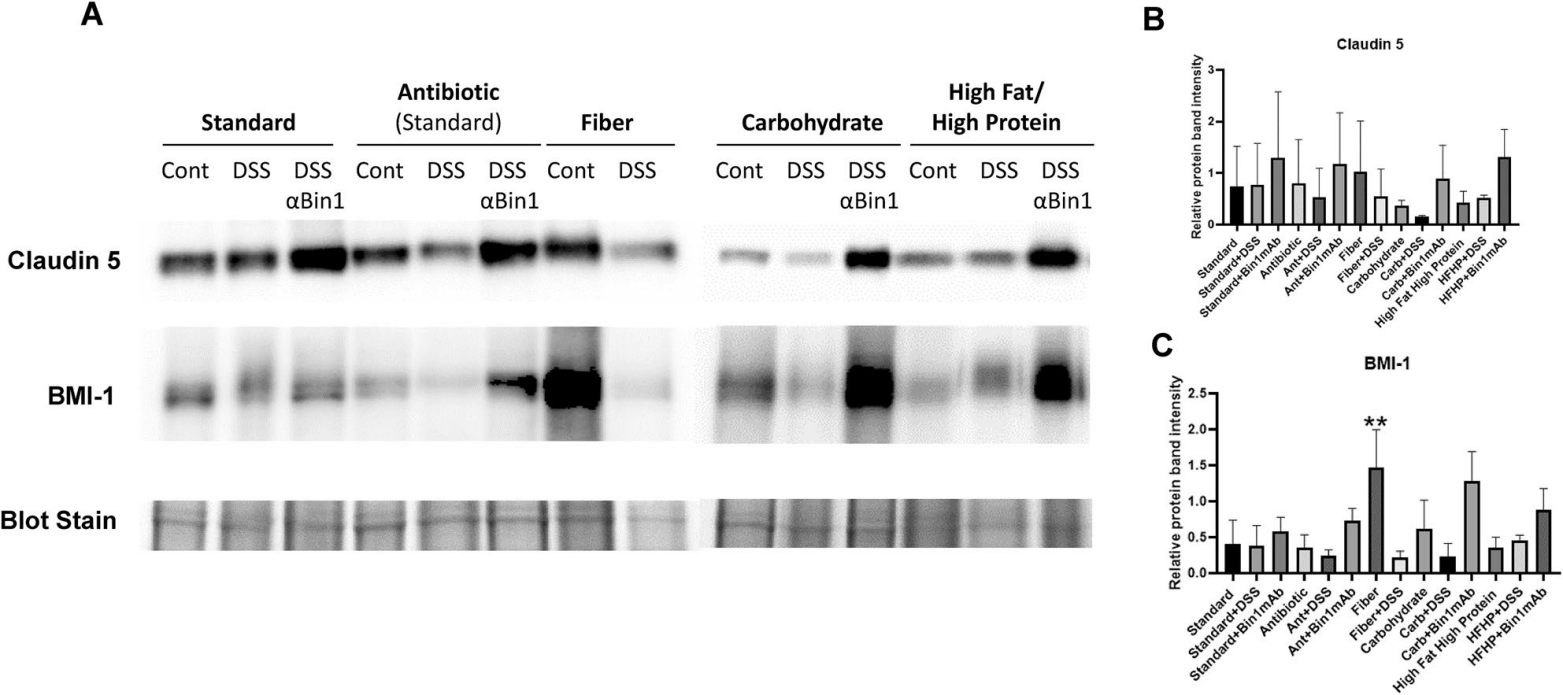
Bin1 mAb treatment increased the expression of the tight junction protein claudin-5 and the stem cell marker BMI-1 of the colon. (**A**) Mice were fed with different diets and induced UC with DSS (n = 5 mice per treatment) for 6 days. The animals were fed drinking water for 24 h and later subjected to Bin1 immunotherapeutic treatment. All the mice fed with fiber diet died in 4 days after Bin1 immunotherapy. The colon of mice was removed 7 days after Bin1 immunotherapy followed by western blotting. Experiments were done in duplicate as samples were highly sensitive to degradation. (**B**) Relative protein band intensity of Claudin 5 and BMI1 (**P < 0.01, as determined by one-way ANOVA).

We demonstrated previously that Bin1 mAb 99D immunotherapy prevents DSS-induced UC in large part by preserving intestinal barrier function^1,2^. Since this effect is associated with increased colonic expression of the tight junction protein claudin-5, we compared the effect of diet on this beneficial effect of Bin1 mAb 99D treatment. Consistent with previous observations, claudin-5 was elevated by administration of the Bin1 mAb after DSS treatment in the GI tract of mice on the standard, antibiotic, carbohydrate and high fat/high protein diets (Fig. 3A). Densitometric data confirmed the high expression of claudin-5 after Bin1 mAb treatment (Fig. 3B). These results confirmed previous observations that Bin1 blockade tightened the intestinal barrier function in a manner associated with claudin-5 induction. In contrast, we observed that the fiber diet rendered the Bin1 mAb immunotherapy post-DSS exposure highly toxic, when administered via the same route and dose as the other dietary cohorts (preventing analysis of gene expression in the fiber cohort treated with DSS + Bin1 mAb 99). This finding was consistent with clinical evidence that high fiber diets exacerbate GI disorders and also render gut-directed immunotherapies highly toxic to the colon.

Since we had observed previously that the benefits of Bin1 mAb immunotherapy are quite robust in preventing or treating DSS-induced UC, we speculated that there may be a positive effect of Bin1 mAb administration on colonic stem cell function in helping preserve normal gut structure (which is otherwise destroyed by inflammatory DSS treatment). On the standard diet, without or with antibiotic present, we observed no effect of the immunotherapy on expression of BMI-1, a GI stem cell transcription factor that is a master regulator of colon structure. However, this was not the case in mice fed a high carbohydrate or high fat/high protein diet, where we observed BMI-1 to be strongly upregulated by Bin1 mAb 99D administration post-DSS treatment (Fig. 3A). Densitometric data confirmed the significant high expression of BMI-1after Bin1 mAb treatment (Fig. 3C) (**P < 0.01, as determined by one-way ANOVA). This observation suggested that diet may be relevant to the impact of Bin1 mAb immunotherapy. In this experiment, we noted that a fiber diet was strongly associated with BMI-1 expression (Fig. 3A). Due to the toxicity of Bin1 mAb immunotherapy in this setting, we could not examine any correlated effect on BMI-1, however, we noted that DSS treatment that powerfully induces UC was associated with a great diminution of BMI-1 expression. Taken together, the results indicated no effect of diet on gut tight junction function, but a significant apparent effect on BMI-1 expression is suggestive of an effect on GI stem cell function.

### Diet influences gut microbiome in UC and its response to Bin1 mAb immunotherapy

**Figure 4:**
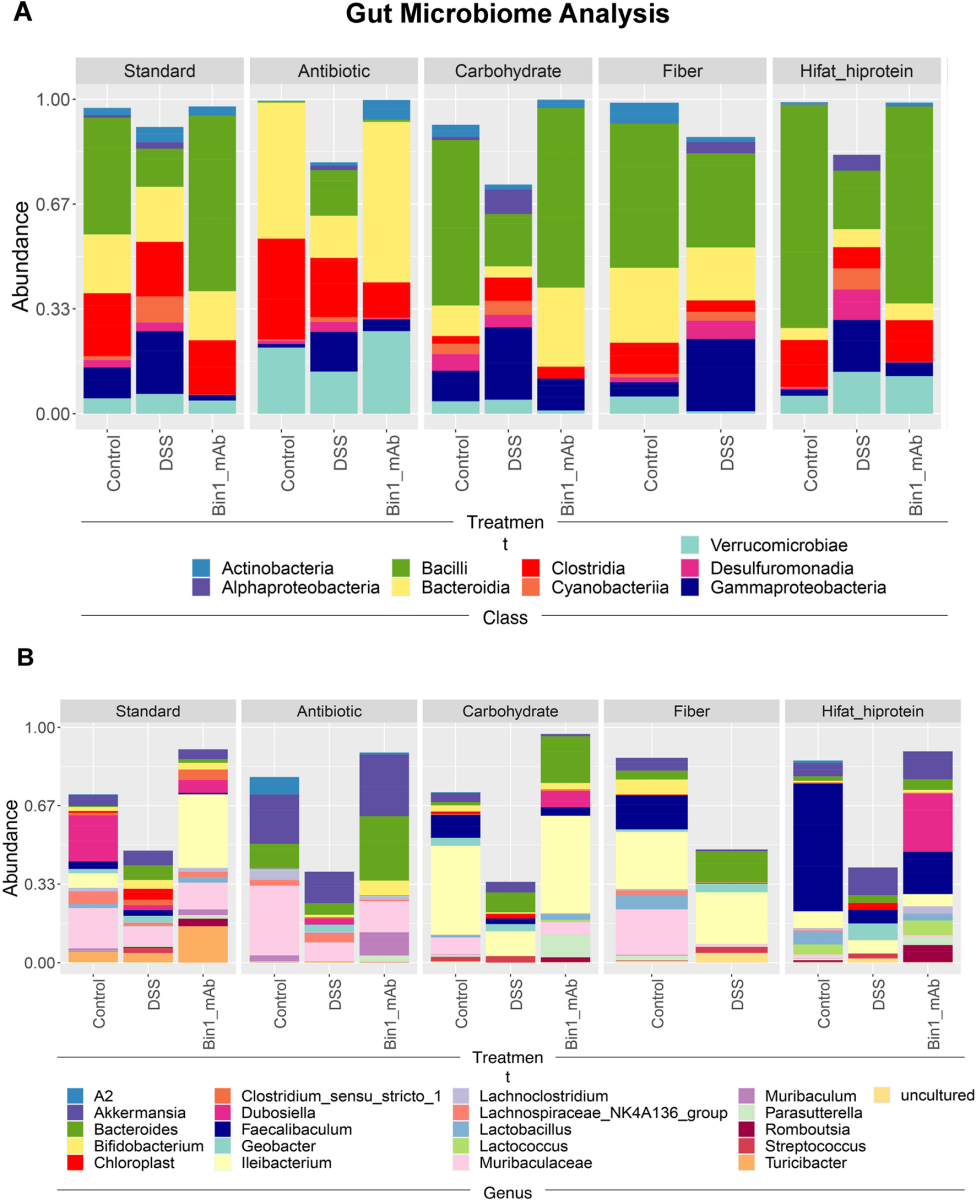
The relative abundance of the microbiome of the fecal pellets of mice from different diet conditions. (**A**) Class *Bacilli* are almost absent upon treatment with antibiotics. (**B**) bacteria of the genus *Faecalibaculum* is abundant in mice fed with a high fat/high protein diet.

We analyzed the class of the gut microbiome fed various diets before or after DSS-induced UC and without or with Bin1 mAb 99D treatment (Fig. 4A). On the standard diet, *Bacilli* decreased during UC induction whereas Alphaproteobacteria, Gammaproteobacteria and Cyanobacteria increased. These patterns were replicated on all the specialized diets. In mice fed the standard diet with antibiotics, there was an absence of Bacilli before and after treatment with Bin1 mAb; however, *Bacilli* re-emerged after UC induction when antibiotics were removed from drinking water. Antibiotic treatment also increased levels of *Bacteriodia* and *Verrucomicrobiae* compared to other diets. The high fat/high protein diet reduced levels of Actinobacteria and *Bacteroidia* but elevated levels of Bacilli. Notably, we observed that treatment with Bin1 mAb 99D reverted gut microbiomes to control patterns on each diet, consistent with the preservation of gut integrity by this immunotherapy (Fig. 4A).

We also analyzed the genus of the gut microbiome on various diets and treatments (Fig. 4B). UC induction in all diets lowered levels of genus *Dubosiella, Ileibacterium*, and *Faecalibaculum*. *Muribaculaceae* (aka family S24-7), which includes undefined genera^23^, was also lowered after UC induction on all diets. The high fat/high protein diet exhibited little *Muribaculaceae* (Fig. 4B). This genus is a major constituent of the gut microbiome that is increased by high-calorie diets, producing enzymes that degrade complex carbohydrates including fiber^24^. *Muribaculaceae* bacteria synthesize the healthful SCFA propionate^25^. On the antibiotic-containing standard diet, *Ileibacterium* were eliminated while levels of bacteria of the genus *Akkermansia, Bacteroides, and Muribaculum* increased. In contrast, the high fat/high protein diet induced high levels of *Faecalibaculum* and *Lactococcus* compared to other diets. Notably, we observed that on the standard diet Bin1 mAb administration increased *Ileibacterium* (a Bacillota increased by high fat diet) and *Turicibacter* (a Bacillota associated with colitis), whereas it reduced *Dubosilla* (a Gram-positive genus reduced by high-fat diets). Alternately, on either the high carbohydrate or high fat/high protein diets, Bin1 mAb administration increased *Dubosilla* and *Bacterioides*.

### Long-term study of diet on gut and muscle/nerve physiology

**Figure 5:**
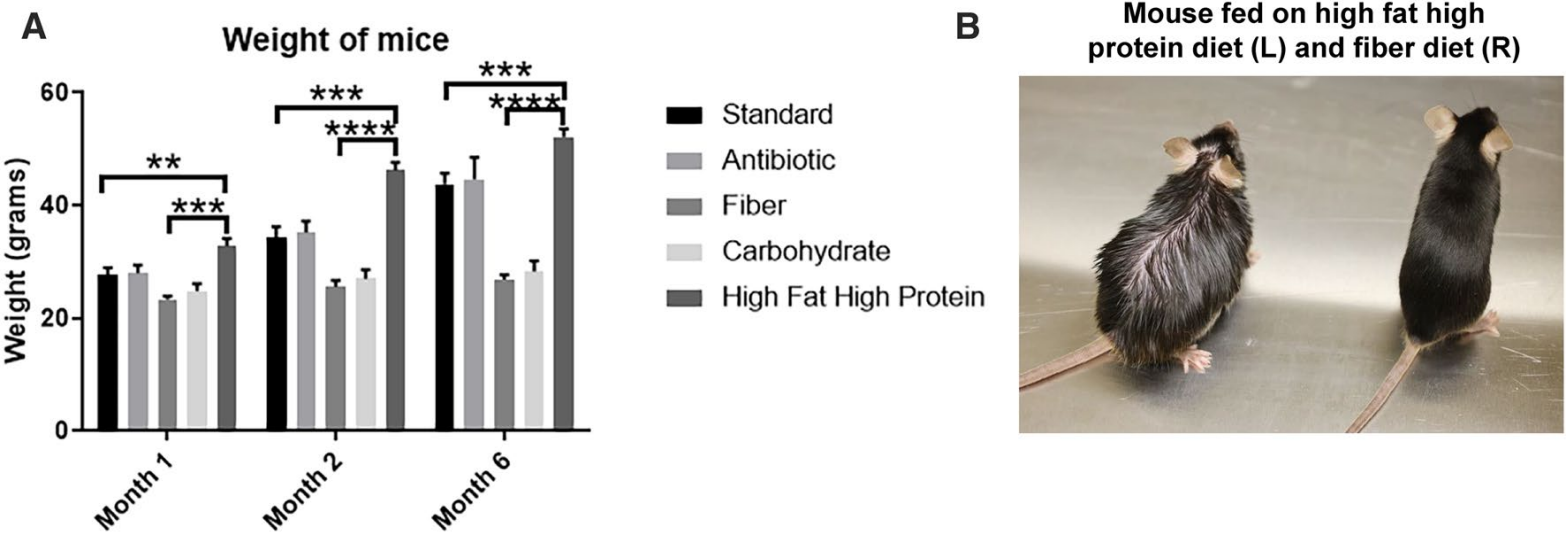
Phenotypic effects of diet on mice with time. Mice were fed with different diets for different time period; they were weighed frequently. (**A**) Weight of mice at months 1, 2 ad 6. (**B**) There was a significant increase in weight of mice after treatment with a high fat/ high protein diet compared to standard diet and fiber diet (**P < 0.01, ***P < 0.001, ****P < 0.0001 as determined by t test). All results are expressed as Mean ± SD; (n = 5 mice per treatment).

There is limited information available on the long-term effects of the various diets studied here on gut or nerve physiology. Accordingly, we monitored several parameters of these physiologies in mice fed various diets over a period of 7 months. There was also a significant difference in weight gain in the high fat/high protein diet compared to the standard diet in months 1 to 6. Mice on high fiber or high carbohydrate diets did not increase significantly in weight over time, compared to mice on the other diets, with the high fat/high protein diet driving the most weight gain and the high fiber diet driving the least gain. (Fig. 5). Overall, the animals on a high fat/high protein diet significantly gained weight in months one (*P* < 0.01), two (*P* < 0.001) and six (*P* < 0.001) compared to animals on stand diet (Fig. 5A). By month six, mice on a high fat/high protein diet were double the size of mice on a fiber diet (Fig. 5B).

**Figure 6:**
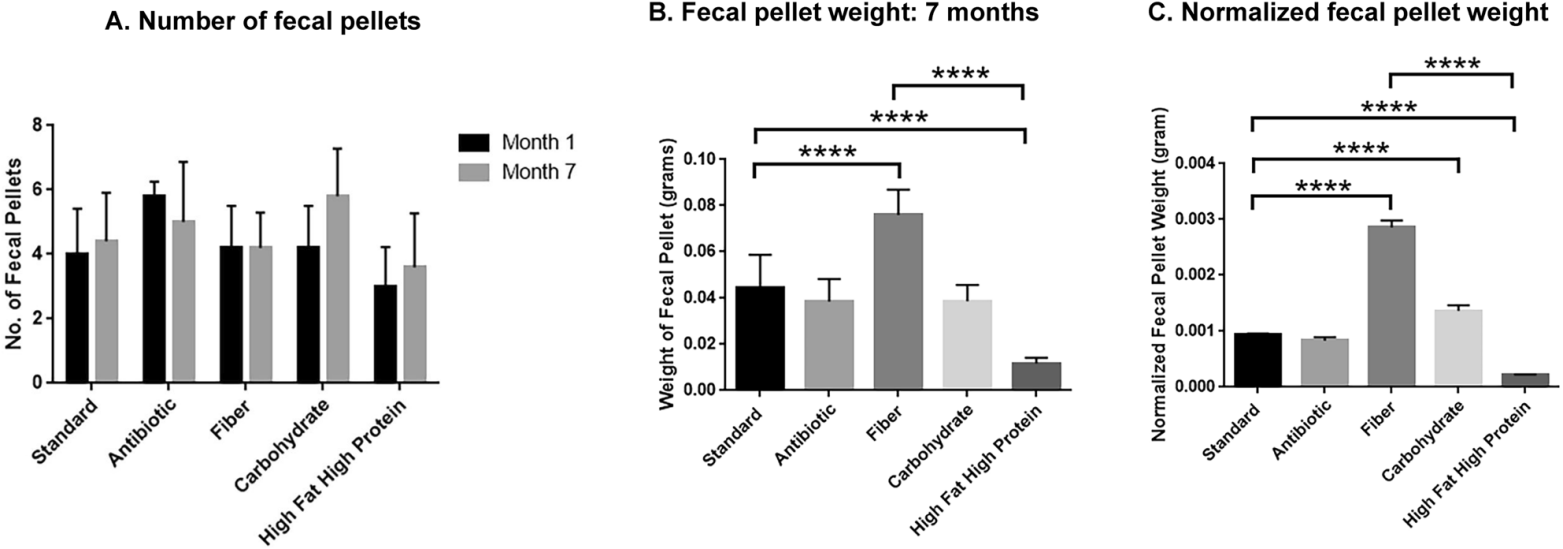
Character of fecal pellets on different diets. Mice were fed with different diets for different time period. They were kept on a raised platform for 10 min and analyzed for: (**A**) Number of fecal pellets, (**B**) Weight of fecal pellets on month 7, (**C**). Normalized weight of fecal pellet. (****P < 0.0001 as determined by t test). All results are expressed as Mean ± SD; (n = 10 fecal pellets per treatment).

In previous studies of Bin1 mAb immunotherapy, we demonstrated that the number of fecal pellets increased during immunotherapy^3^. Here we observed that mice on a high fat/high protein diet produced relatively fewer fecal pellets than mice on other diets in months 1 and 7 (Fig. 6A). The weight of fecal pellets produced by mice on a high fat/high protein diet was significantly lower than those produced by mice on other diets (*P* < 0.0001) (Fig. 6B). Normalization of the fecal pellet to body size of mice also demonstrated that the fecal pellet of mice on a high fat/high protein diet had significant smaller size compared to the fecal pellet on other diets (*P* < 0.0001) (Fig. 6C).

**Figure 7:**
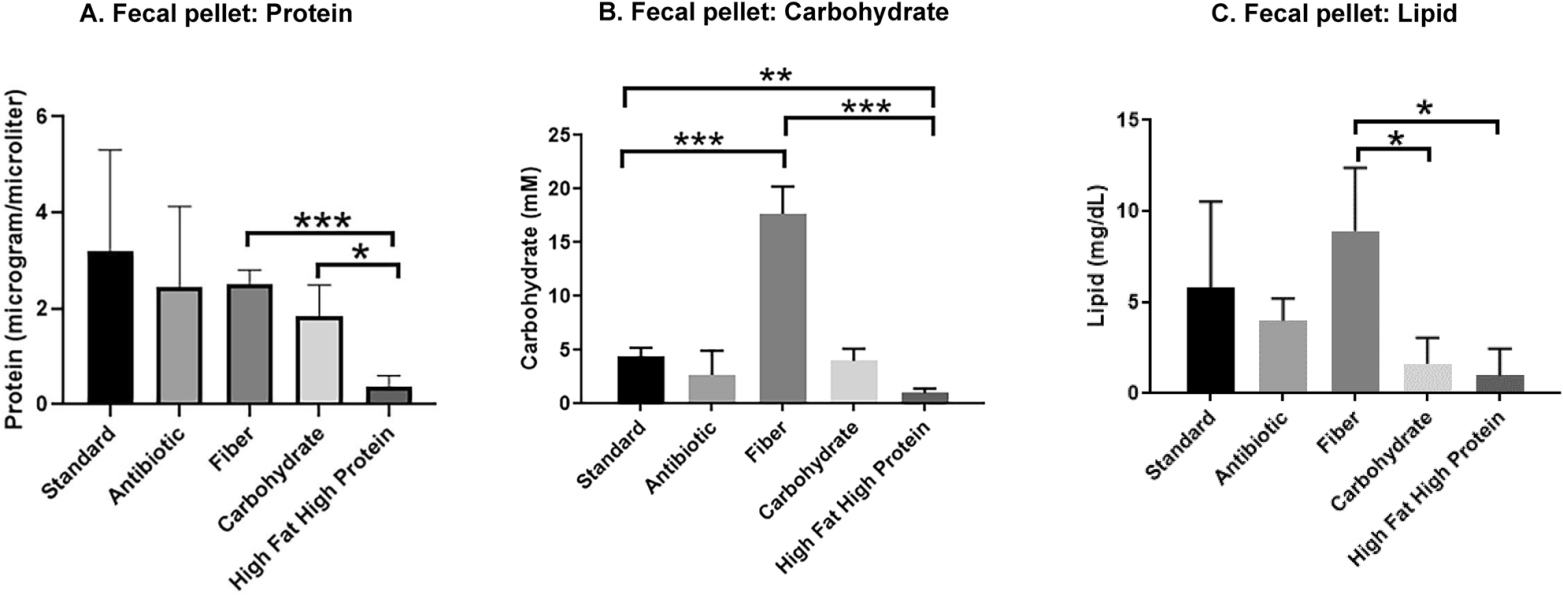
Poor nutrient content of fecal pellets from mice on a high fat/high protein diet. Mice were fed with different diets for three months and the nutrient content of the fecal pellets analyzed. Mice on a high fat/high protein diet had low levels of (**A**) Protein, (**B**) Carbohydrate and (**C**) Lipids. (*P < 0.05, **P < 0.01, ***P < 0.001, ****P < 0.0001 as determined by t test). All results are expressed as Mean ± SD; (n = 5 mice per treatment).

To determine nutrient content that remained in fecal pellets, we determined the protein (Fig. 7A), carbohydrate (Fig. 7B) and lipid (Fig. 7C) contents on different diets. Notably, pellets from mice on a high fat/high protein diet exhibited the lowest amount of these nutrients compared to that of mice on other diets (protein and carbohydrate: *P* < 0.001, fiber vs high fat/high protein; lipid: *P* < 0.05, fiber vs high fat/high protein), whereas pellets from mice on a high fiber diet had the highest amount of nutrients remaining (Fig. 7A–C).

**Figure 8:**
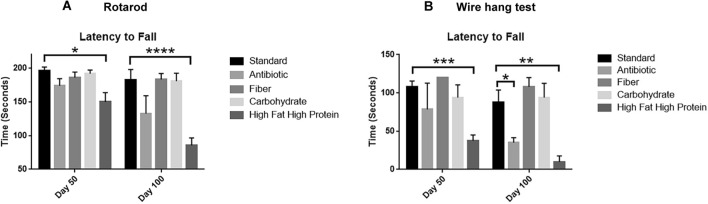
Mice on a high fat/high protein diet exhibit muscle weakness. Mice on different diets were subjected to rotarod and wire hang test at different periods. (**A**) Mice on antibiotics and a high fat/high protein diet had the latency to fall quickly from a rotarod. (**B**). Mice on antibiotics and a high fat/high protein diet had the latency to fall quickly in a wire hang test. (*P < 0.05, **P < 0.01, ***P < 0.001, ****P < 0.0001 as determined by t test). All results are expressed as Mean ± SD; (n = 5 mice per treatment).

To assess muscle coordination and strength, we regularly tested mice fed different diets on a standard rotarod balance test and a wire-mesh hanging test. Mice fed a high fat/high protein diet performed by far the most poorly on both tests. In days 50 (*P* < 0.05) and 100 (*P* < 0.0001), mice fed with a high fat/high protein diet performed significantly poorly on a rotarod compared to the standard diet. Mice fed with antibiotics also performed significantly poorly on the wire-mesh hanging test (*P* < 0.05, Day 100). Mice fed a high fiber diet tended to perform somewhat better than mice fed other diets in both rotarod and wire hang tests (Fig. 8A, B). While we did not characterize the basis for loss of balance and muscle strength evaluated by these tests, the data suggested that mice fed a high fat/high protein diet were weaker than could be explained by their slightly heavier weight, suggesting the possibility of age-associated muscle or nerve deficiencies.

**Figure 9:**
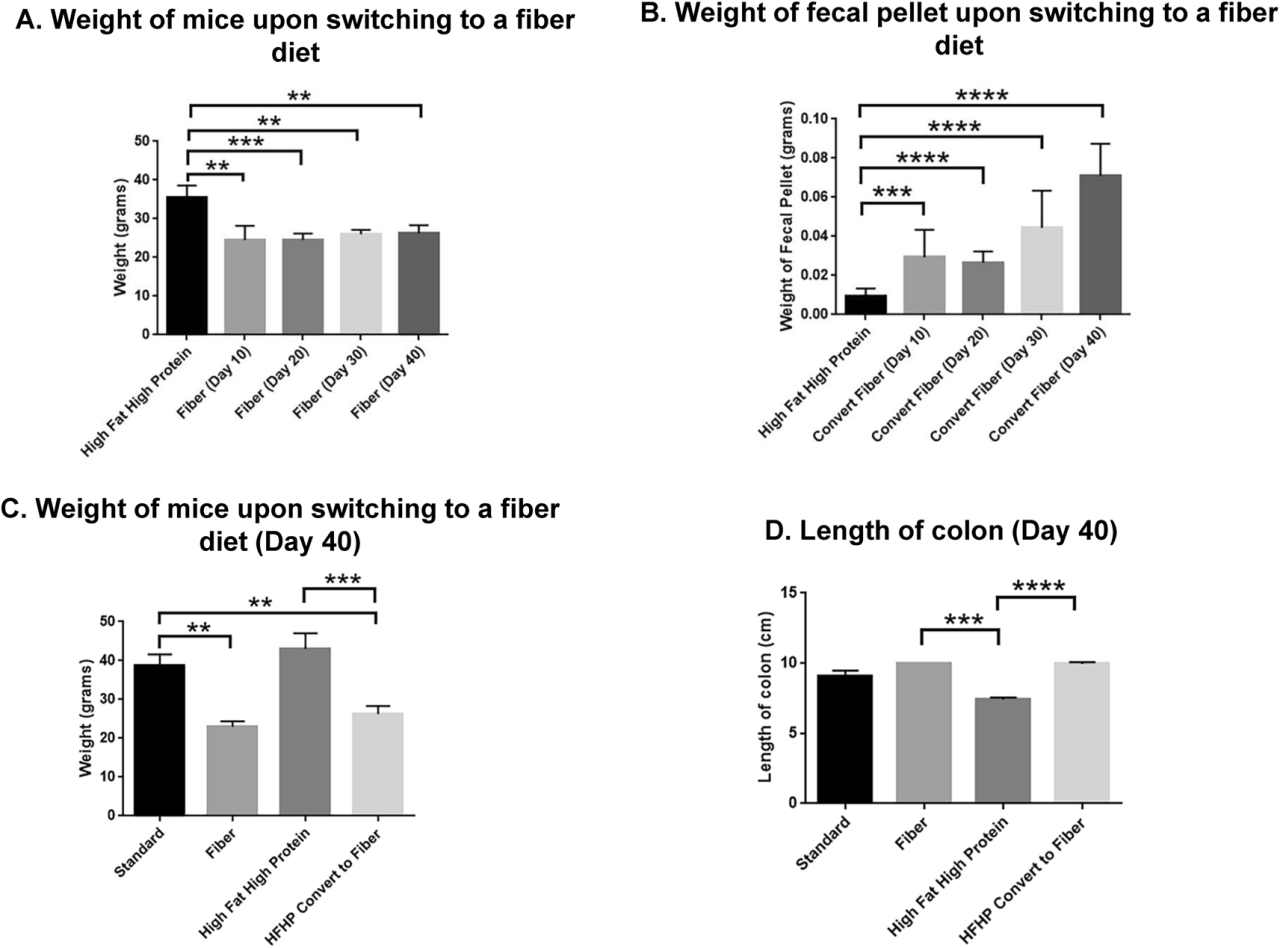
Effect of diet switching from a high fat/high protein diet to a high fiber diet. Mice were fed with a high fat/high protein diet for two months and switched to a fiber diet for 40 days. (**A**) The weight of the animals decreased upon switching to a fiber diet. (**B**) The weight of fecal pellets increased upon switching to a fiber diet. (**C**) Comparison of weight of animals on different diets 40 days after switching to a fiber diet. (**D**) The length of colon increased after switching to a fiber diet. The colon was harvested 40 days after switching from a high fat/high protein diet to a fiber diet. (**P < 0.01, ***P < 0.001, ****P < 0.0001 as determined by t test). All results are expressed as Mean ± SD (n = 5 mice per treatment).

**Figure 10:**
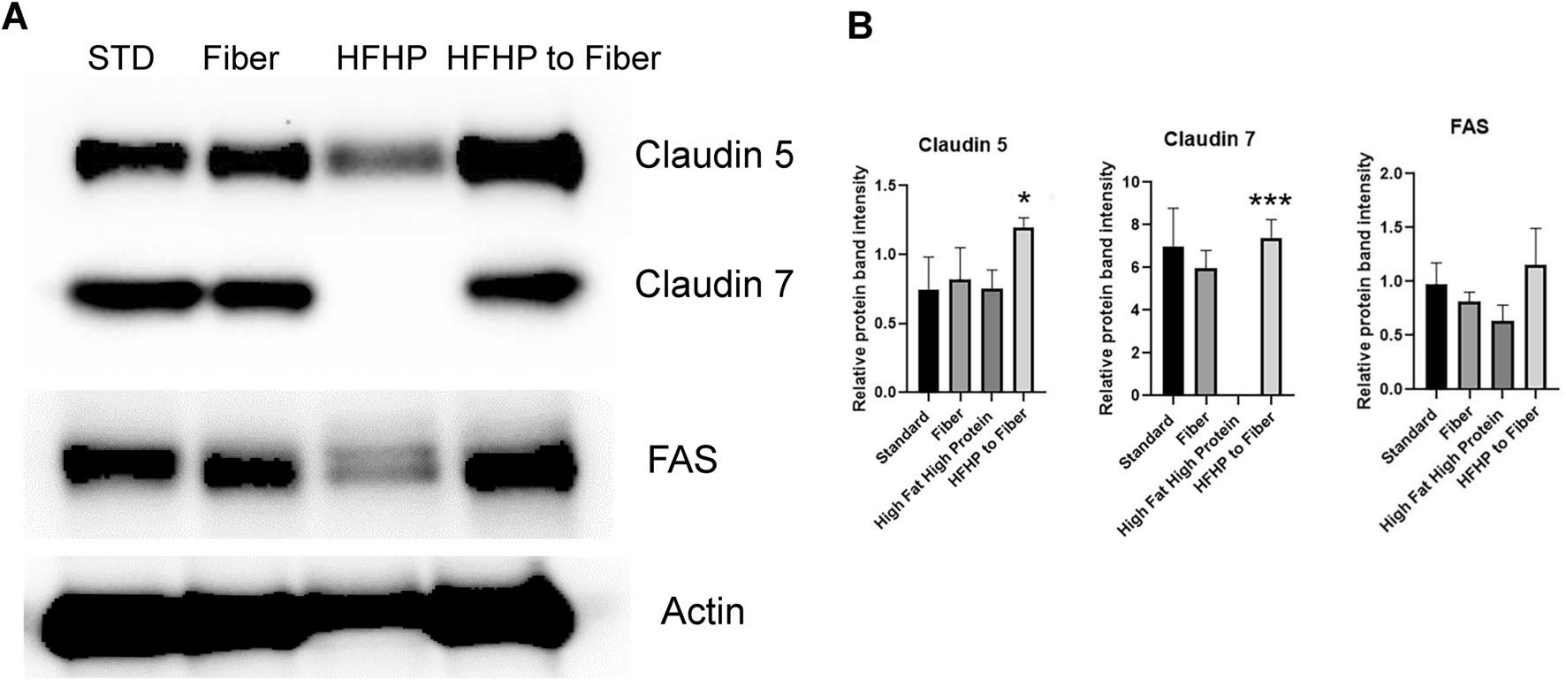
Expression of claudin-5/7 and Fas in the colon are altered by switching to a high fiber diet. (**A**) A high fat diet decreased the expression of claudin-5, claudin-7 and fas; whereas, switching to a fiber diet increased its expression, as determined by western blot. Each blot is the representative of three blots. (**B**) The relative protein band intensity of claudin-5 (*P < 0.05), claudin-7 (***P < 0.001) and Fas determined by Image J and statistically analyzed by one-way ANOVA (n = 3).

To gain initial insight into how diet changes could limit obesity in mice fed a high fat/high protein diet, mice started on this diet for 60 days were switched to a fiber diet and monitored for 40 additional days. Mouse weight decreased significantly by day 10 after switching to a fiber diet (*P* < 0.01) (Fig. 9A). The weight loss remained the same until day 40. The fecal pellets produced by the mice after switching from a high fat/high protein diet to a fiber diet significantly increased in weight by 10 days (*P* < 0.001) and continued to increase to 40 days (*P* < 0.0001) (Fig. 9B). We compared the weight of mice on different diets on day 40. There was a significant decrease in weight loss in mice switched from a high fat/high protein diet to a fiber diet when compared to mice on a high fat/high protein diet (*P* < 0.001). The mice switched from a high fat/high protein diet to a fiber diet had similar weight to mice fed with fiber diet (Fig. 9C). The colon lengths also increased significantly after switching to a fiber diet (*P* < 0.0001), suggestive of a more healthful colon (Fig. 9D). We monitored the expression of the tight junction proteins claudin-5 and claudin-7, another claudin that increases intestinal barrier function, during the transition of these animals. On a high fat/high protein diet, levels of each claudin were reduced, whereas switching from that diet to a high fiber diet restored claudin levels to those expressed in the colon on standard or high fiber diets. We also found that a high fat/high protein diet reduced levels of Fas/CD95, a cytokine receptor known to prevent colon tumorigenesis^26^ and switching to a fiber diet also restored Fas/CD95 expression like claudins-5/7 (Figs. 10A, S2). Densitometric analysis confirmed significantly high expression of claudin-5 (*P < 0.05) and claudin-7 (**P < 0.001) in the colon tissues of mice switched to a fiber diet. The expression of Fas was also high in the colon after switching to a fiber diet (Fig. 10B). Taken together, these data provide new knowledge about how mice respond to different dietary conditions, short or long term, helping establish a foundation for further study in mouse models of how diet affects gut physiology and immunotherapy.

### Bin1 immunotherapy influences colon length

**Figure 11:**
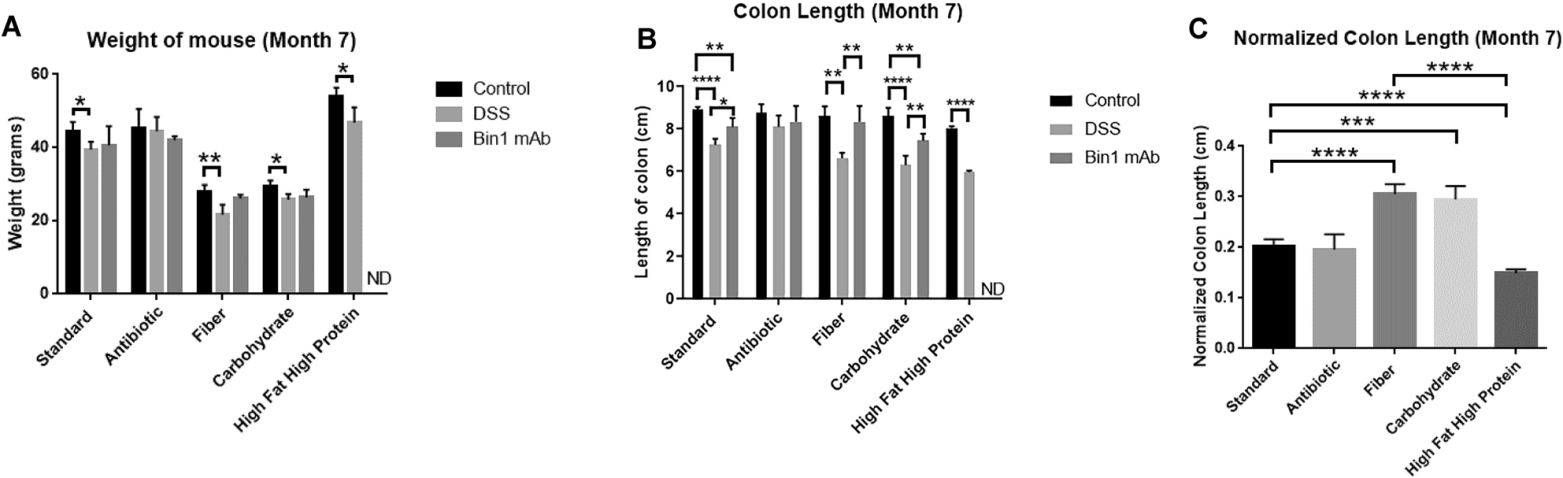
Long-term effects of diet on UC induction and Bin1 immunotherapy. Mice on different diets for 7 months were induced UC using 3% DSS. After 6 days, they were treated with Bin1 mAb. Mice on a high fat/high protein diet succumbed to UC. (**A**) Weight of mice, (**B**) Length of colon, (**C**) Normalized colon length. (*P < 0.05, **P < 0.01, ***P < 0.001, ****P < 0.0001 as determined by t test). All results are expressed as Mean ± SD; (n = 5 mice per treatment).

Colon length is correlated with GI stress. In mice fed different diets for 7 months, we noticed that the animal weight varied with diet (Fig. 11A). Induction of UC with DSS decreased the weight of mice in all the treatments, with significant weight decrease (*P* < 0.05) in animals on standard, carbohydrate and high fat high protein diets. Treatment with Bin1 mAb increased the weight in mice on standard and fiber diets. UC induction markedly reduced the length of the colon, whereas beneficial treatment with Bin1 mAb 99D significantly helped recover normal colon length in mice on standard (*P* < 0.05), fiber (*P* < 0.01) and carbohydrate diets (*P* < 0.01) (Fig. 11B). Notably, mice fed a high fat/high protein diet had significantly small colon and were most sensitive to DSS treatment as illustrated by colon shortening (*P* < 0.0001) (Fig. 11B and C). This diet also rendered the Bin1 mAb immunotherapy toxic, preventing completion of the analysis among mice in the DSS + Bin1 mAb arm of the experiment. Similarly, mice on a fiber diet were very sensitive to DSS-induced UC, with all of them expiring within 3–4 days of treatment. Prolonging immunotherapy by 7 days rescued the animals on a fiber diet; nevertheless, they had loose stool. Overall, these results showed that a high fat/high protein diet increased sensitivity to DSS-induced UC and was deleterious to Bin1 mAb immunotherapy. Furthermore, they showed that a high fiber diet increases the severity of DSS-induced UC, but that even this toxic modifier effect could be rescued by prophylactic treatment with Bin1 mAb 99D.

### Bin1 immunotherapy induces short chain fatty acids

**Figure 12:**
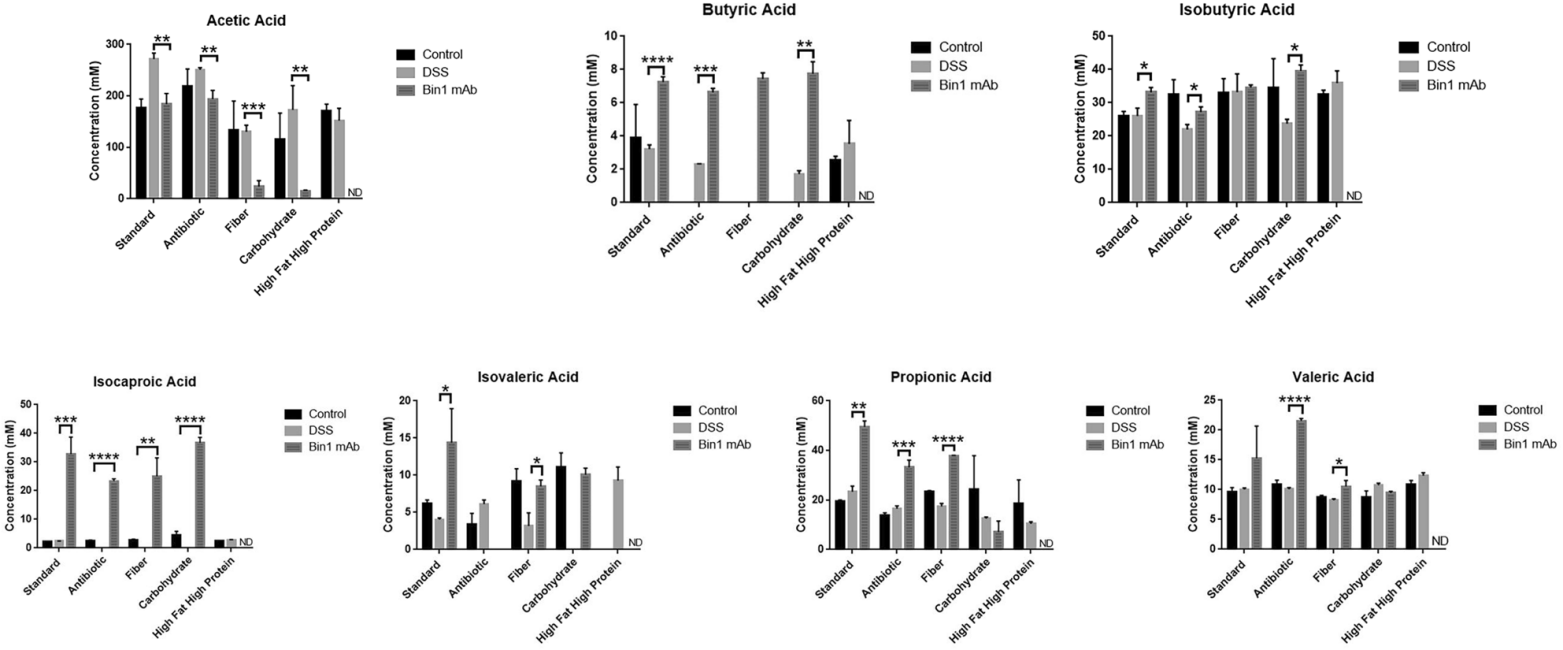
Metabolome comparisons of short chain fatty acids (SCFAs) after UC induction and Bin1 immunotherapy. Mice on different diets for 7 months were induced UC using 3% DSS. After 6 days, they were treated with Bin1 mAb. The fecal pellets collected after 7 days were cultured anaerobically for 3 days and the metabolome determined. Bin1 mAb treatment lowered the levels of acetic acid that is known to increase during UC. Bin1 mAb treatment increased the levels of butyric acid, isobutyric acid, isocaproic acid, isovaleric acid, propionic acid and valeric acid either in all the diets or some diets (**P < 0.01, ***P < 0.001, ****P < 0.0001 as determined by t test). All results are expressed as Mean ± SD; (n = 4 mice per treatment).

Recently we showed that the beneficial effects of passive immunotherapy with Bin1 mAb 99D in preventing and treating UC was based to significant extent in maintaining a robust intestinal barrier function in colonic epithelia, thereby staving off DSS-induced UC^1,2^. Short chain fatty acids (SFCA) are also known to tighten intestinal barrier functions^27^. Therefore, we examined SFCA levels in the gut metabolome of mice that were fed different diets before or after UC induction, without or with Bin1 mAb treatment (Fig. 12) (Supplementary Table 1). High level of acetic acid is a hallmark during UC. Induction of ulcerative colitis with DSS increased the expression of acetic acid in mice fed with standard, antibiotic and carbohydrate diets. Bin1 mAb administration was observed to significantly limit levels of gut-borne acetic acid (*P* < 0.01, standard, antibiotic, carbohydrate diets; *P* < 0.001, fiber diet).

Bin1 mAb administration acted in all diets to significantly increase levels of the beneficial SFCAs butyric acid and isocaproic acid, which were found at low levels in all diets except the standard diet. Propionic acid was similarly significantly increased in all diets except the high carbohydrate diet. Elevations in isobutyric, isovaleric, and valeric acid were also seen in certain diets (Fig. 12). Thus, Bin1 immunotherapy appeared to generally increase SCFAs in animals protected against UC, suggesting they could connect the pleiotropic effects of Bin1 mAb 99D administration on intestinal barrier function, anti-inflammatory signaling, ENS status and healthful microbiome status in mediating a beneficial UC treatment.

## Discussion

The factors responsible for the development of UC are not yet fully understood, but diet is certainly one risk factor. In particular, a western diet rich in high fat/high protein diet is considered a significant risk factors for UC^28–30^. Animal models confirm that a high fat/high protein diet increases UC risk^31–33^. In addition, high fat/high protein diets may contribute simultaneously to obesity and psychiatric disorders by suppressing hippocampal brain-derived neurotrophic factor expression with the disturbance of gut microbiota composition^34^.

We performed a short-term (one month) and long-term study (seven months) to determine whether diet influences the health of the colon. Since diet influences the efficiency of immunotherapeutic drugs, we also determined whether the diet influences the efficiency of Bin1 mAb during UC treatment. The findings of this study provide a foundation for investigations in mouse models of the effect of diet on UC immunotherapies.

Our study demonstrated that a diet rich in fat and proteins negatively influenced the health of the colon. We did not observe induction of UC as a result of a high fat/high protein diet delivered over a course of up to 7 months. However, we observed that this diet served as a positive modifier of inflammation in the setting of UC induction and also Bin1 immunotherapy administration. This diet also promoted a gut physiology associated with reduced expression of tight junction proteins that enforce intestinal barrier function; altered the status of enteric neuron, colonic stem cells, and skeletal muscle function; and led to changes in the gut microbiome toward a more dysbiotic state—all hallmarks of UC and other IBD. As a result of these findings, we suggest nomenclature for a “hunger-like condition” (peinosis) [peina (Greek), hunger; osis (Greek), condition] that is characterized by production of low-nutrient (low-energy) fecal pellets generated in the colon as a result of a high fat/high protein diet—one which may create a condition that modifies gut inflammation or the effects of UC immunotherapy to increase toxicity^35^. In comparing features of standard diet to a high fat/high protein diet, we found the latter to be more characterized by peinosis and signs of poor health. Overall, the results suggested that a high fat/high protein diet is deleterious for health and not recommended during UC immunotherapy.

Antibiotic abuse has led to development of drug resistant bacteria that is a global public health challenge phenomenon^36^. In addition, antibiotics are used in agriculture, aquaculture and animal husbandry to prevent infection and increase yield. The exposure of antibiotic residues from food could lead to the development of antibiotic resistant bacteria that progressively increase mortality from multidrug-resistant bacterial infections, thereby posing a tremendous threat to public health^37,38^. In our studies we observed a marginal increase in colon length in mice provided antibiotics compared to standard diet alone (30 days). The antibiotic supplemented mice also had a dysbiotic microbiome. In this study, we showed that mice on standard diet with antibiotics had muscle weakness compared to mice on standard diet alone. Similar results were documented in athletes on antibiotics^39^. The study demonstrated that antibiotics could influence colon health and should not be used during UC immunotherapy.

High fiber diets are widely viewed as healthful to the gut and brain^40^. One of the chief energy sources in the colon is SCFAs, which are produced in abundance by gut microorganisms feeding on a fiber diet^41^. In our study, mice on a high fiber diet did not increase weight; fecal pellets had the highest nutrient content; and these mice had a good microbiome and motor skills/muscle strength. However, once UC was induced, a high fiber diet was profoundly deleterious to health. We were surprised to find that the robust beneficial effects of Bin1 immunotherapy for UC treatment in the DSS model were converted to highly toxic effects on a fiber diet, unless the administration of the immunotherapy was delayed at least a week after UC induction (upon which surviving animals benefited but had frequent loose stools). While we found that a carbohydrate-rich diet did not negatively impact colon health, or Bin1 immunotherapy response, this diet promotes obesity and type II diabetes and may be less desirable for middle-aged or elderly individuals receiving UC immunotherapy^42^. Overall, a standard diet containing a mixture of lipids, fiber, carbohydrates and proteins was most healthful in the context of UC induction and beneficial Bin1 immunotherapy.

Our study illuminated changes in the gut microbiome and SFCA metabolome that may inform future studies of the impacts of diet on UC immunotherapy. Clinical UC has been characterized by high levels of Gammaproteobacteria^43^, which our preclinical investigations appeared to replicate during UC induction in the DSS mouse model. Overall, UC induction lowered bacterial diversity whereas Bin1 mAb 99D treatment increased bacterial diversity in a manner associated with beneficial treatment^1–3^. With regard to metabolomic changes, Bin1 mAb immunotherapy lowered the levels of acetic acid known to positively influence inflammation, whereas it increased the levels of several healthful SCFA, especially butyric acid and propionic acid on all the diet tests. These SCFAs are known to positively influence intestinal barriers^27^. Thus, Bin1 targeting by the passive immunotherapeutic strategy we are developing may produce treatment benefits in preventive or treatment settings through multiple mechanisms, possibly linked by SCFA production that can promote intestinal epithelial barrier function, influence enteric neuronal and stem cell functions, and normalize the gut microbiome via lowering Bin1 expression^1–3^.

Overall, the study demonstrates that a high fat high protein diet is deleterious since the nutrients are absorbed by the small intestine leaving nothing in the large intestine. Absence of nutrients in the colon causes it to reduce size. As yet we do not know why ulcerative colitis starts in the distal colon/rectum and move to the proximal part. The increased nutrient deficiency in the distal colon could be speculated why ulcerative colitis starts in the distal colon and progress to the proximal site.

In this study we demonstrated the diet influence the quality of immunotherapy. During immunotherapeutic treatment of UC, it is advisable that the physician check and recommend the best diet to the patients. In this study we also showed that the stem cells are positively influenced by Bin1 mAb. Identifying the stem cells and its transformation to mature cells would lead to better treatment strategies for UC.

## Supplementary Information

Supplementary Legends.

Supplementary Figures.

Supplementary Table S1.

Supplementary Table S2.

Supplementary Table S3.

Supplementary Table S4.

## Data Availability

The datasets used and/or analyzed during the current study available from the corresponding author on reasonable request.
