# Supplementary material for: Diet effects on colonic health influence the efficacy of Bin1 mAb immunotherapy for ulcerative colitis
Source: Sci Rep. 2023 Jul 21;13:11802. doi: 10.1038/s41598-023-38830-2 (PMC10361997; doi:10.1038/s41598-023-38830-2)
Supplement: Supplementary file 1 — Supplementary Legends. [file 41598_2023_38830_MOESM1_ESM.docx]

**Supplementary Figures**

**Fig. S1. Figure illustrating the experimental conditions of the study**. Mice were fed with different diets (standard, standard + antibiotic, fiber, carbohydrate, high fat/high protein diets) for one month or seven months. The weight of animals, fecal pellet weight, fecal pellet nutrient content, rotarod test, wire hang test was conducted at regular intervals. At the end of one month or six months the mice were induced UC by DSS. After six days, the mice were fed regular water for 24 hours, followed by treatment with Bin1 mAb that is known to be protective against the disease. The weight, colon length, protein expression, microbiome and metabolome was analyzed 7 days after Bin1 mAb treatment.

**Fig. S2. High fat/high protein diet decreased NeuN expressing enteric neurons in the colon muscularis mucosa.** The muscularis mucosa of the colon of mice treated with standard, antibiotic, fiber and carbohydrate diets had good NeuN expressing enteric neurons compared to colon from mice fed with a high fat/high protein diet.

**Fig. S3**. **Bin1 mAb treatment increased the expression of the tight junction protein claudin-5 and the stem cell marker BMI-1 of the colon**. Mice were fed with different diets and induced UC with DSS (n=5 mice per treatment) for 6 days. The animals were fed drinking water for 24 hours and later subjected to Bin1 immunotherapeutic treatment. All the mice fed with fiber diet died in 4 days after Bin1 immunotherapy. The colon of mice was removed 7 days after Bin1 immunotherapy followed by western blotting. Experiments were done in duplicate as samples were highly sensitive to degradation. Individual blots are shown.

**Fig. S4. Expression of claudin-5/7 and Fas in the colon are altered by switching to a high fiber diet**. A high fat/high protein diet decreased the expression of claudin-5, claudin-7 and fas; whereas, switching to a fiber diet increased its expression, as determined by western blot. The three blots are shown.

**SUPPLEMENTARY TABLES**

**Table S1.** Standard diet ingredients and nutritional profile**.**

**Table S2.** Fiber diet ingredients and nutritional profile.

**Table S3.** Carbohydrate diet ingredients and nutritional profile.

**Table S4.** High fat/high protein diet ingredients and nutritional profile.
