# Supplementary material for: Diet effects on colonic health influence the efficacy of Bin1 mAb immunotherapy for ulcerative colitis
Source: Sci Rep. 2023 Jul 21;13:11802. doi: 10.1038/s41598-023-38830-2 (PMC10361997; doi:10.1038/s41598-023-38830-2)
Supplement: Supplementary file 2 — Supplementary Figures. [file 41598_2023_38830_MOESM2_ESM.pptx]

## Slide 1
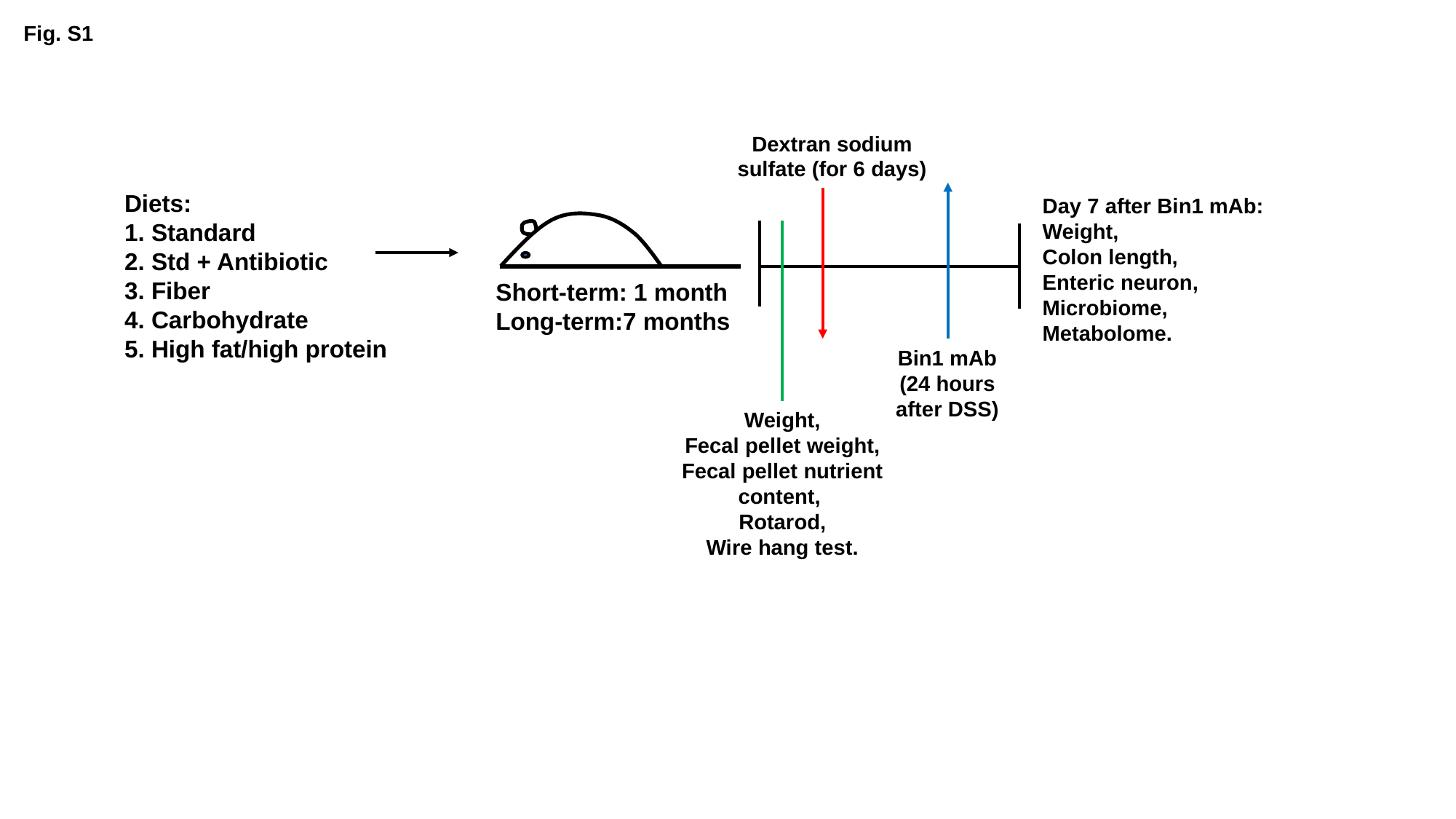

Fig. S1
Dextran sodium sulfate (for 6 days)
Diets:
1. Standard
2. Std + Antibiotic
3. Fiber
4. Carbohydrate
5. High fat/high protein
Day 7 after Bin1 mAb:
Weight,
Colon length,
Enteric neuron,
Microbiome,
Metabolome.
Short-term: 1 month
Long-term:7 months
Bin1 mAb (24 hours after DSS)
Weight,
Fecal pellet weight,
Fecal pellet nutrient content,
Rotarod,
Wire hang test.

## Slide 2
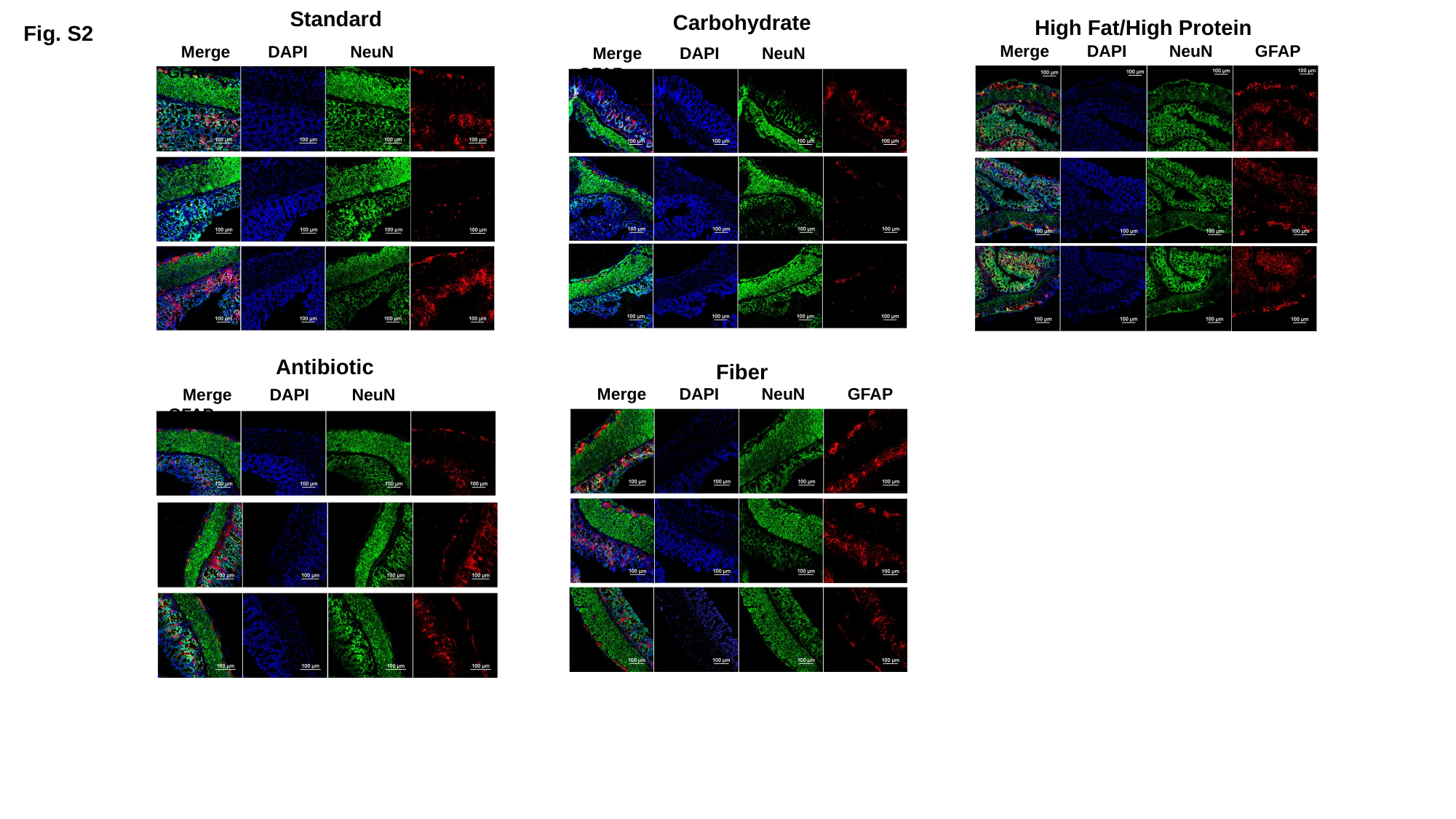

Standard
Carbohydrate
High Fat/High Protein
Fig. S2
 Merge DAPI NeuN GFAP
 Merge DAPI NeuN GFAP
 Merge DAPI NeuN GFAP
Antibiotic
Fiber
 Merge DAPI NeuN GFAP
 Merge DAPI NeuN GFAP

## Slide 3
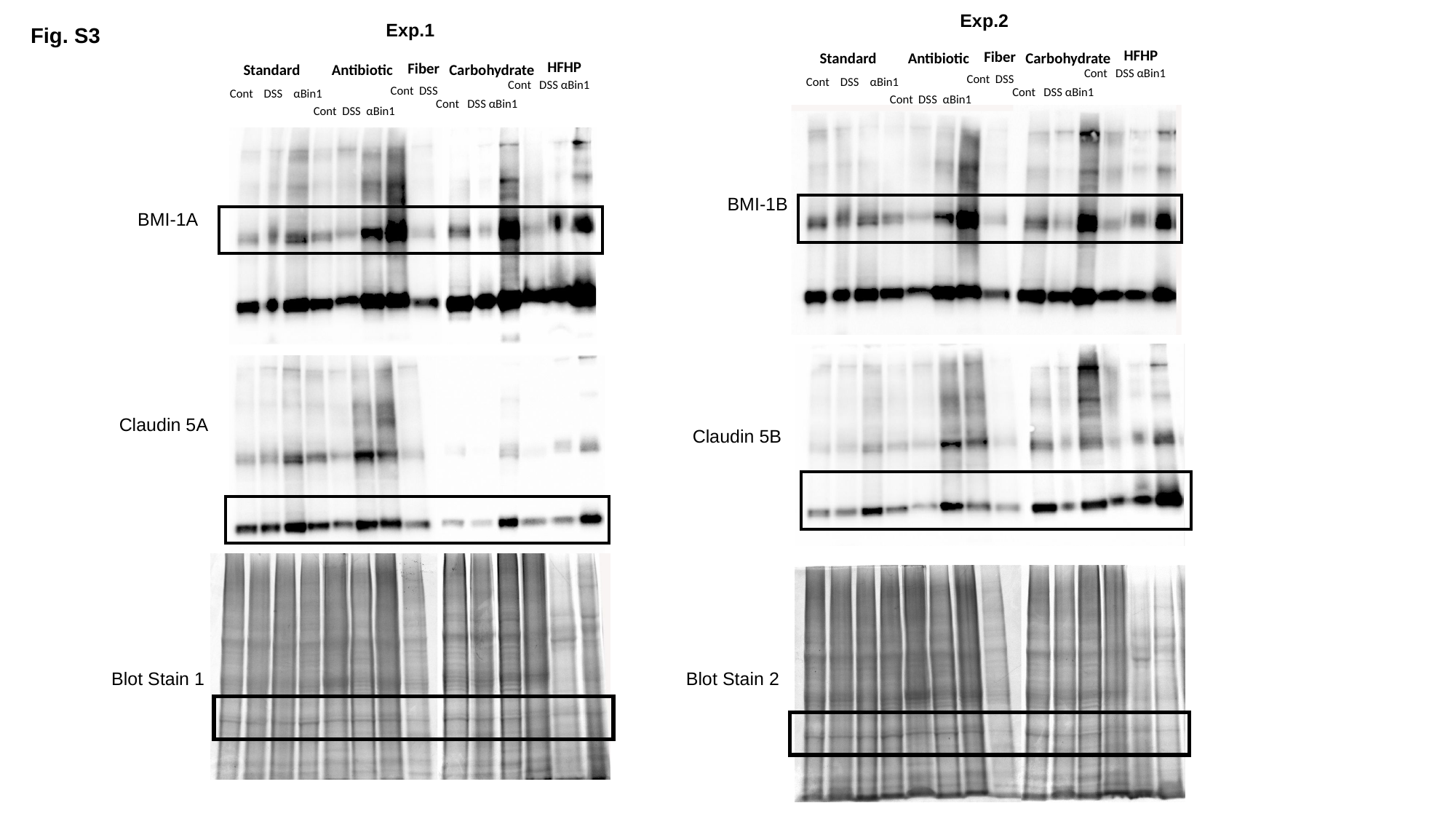

Exp.2
Exp.1
HFHP
Fiber
Carbohydrate
Standard
Antibiotic
Cont DSS αBin1
Cont DSS
Cont DSS αBin1
Cont DSS αBin1
Cont DSS αBin1
HFHP
Fiber
Carbohydrate
Standard
Antibiotic
Cont DSS αBin1
Cont DSS
Cont DSS αBin1
Cont DSS αBin1
Cont DSS αBin1
BMI-1B
BMI-1A
Claudin 5A
Claudin 5B
Blot Stain 2
Blot Stain 1
Fig. S3

## Slide 4
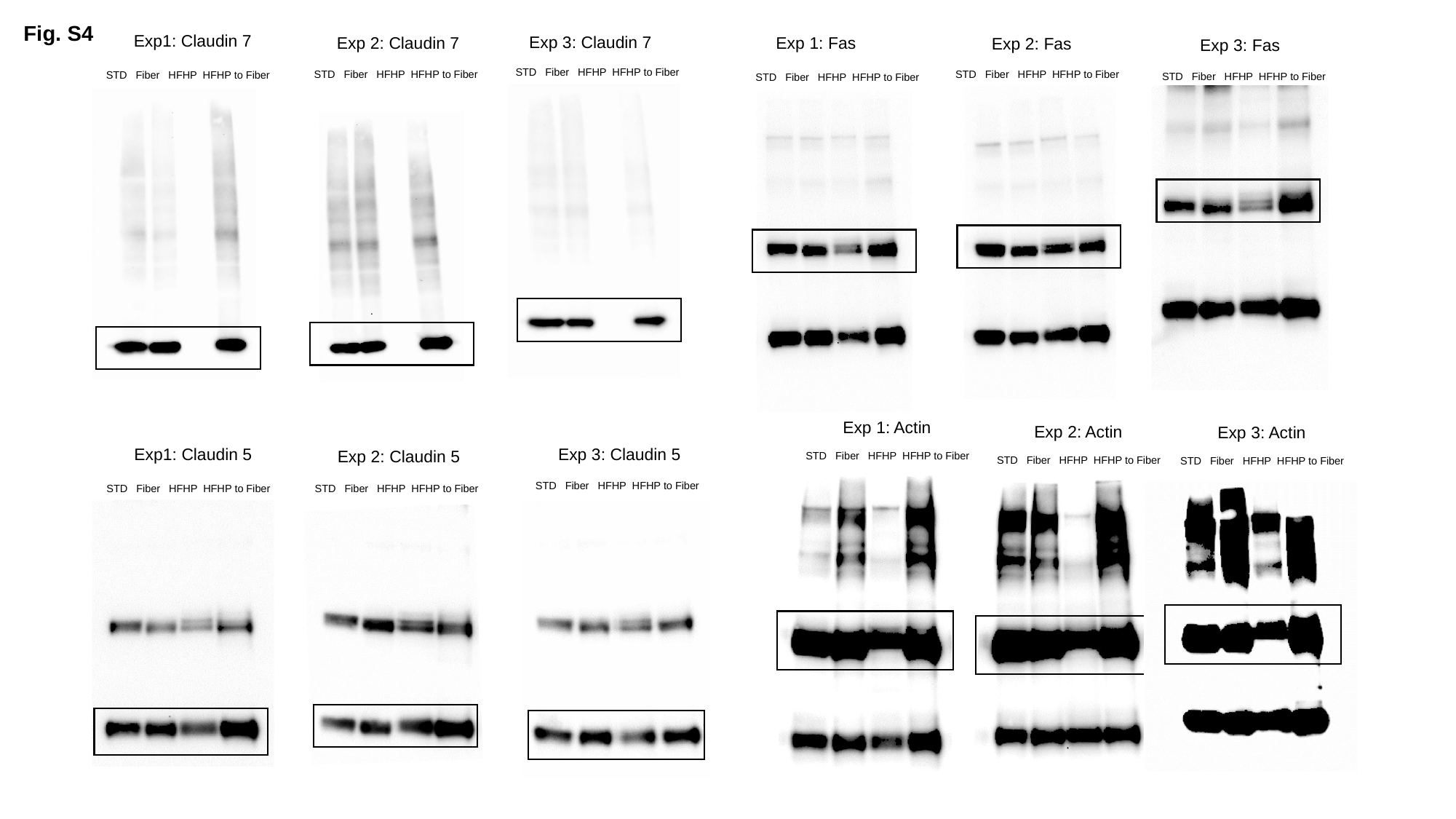

Fig. S4
Exp1: Claudin 7
Exp 3: Claudin 7
Exp 2: Claudin 7
 STD Fiber HFHP HFHP to Fiber
 STD Fiber HFHP HFHP to Fiber
 STD Fiber HFHP HFHP to Fiber
Exp 1: Fas
Exp 2: Fas
Exp 3: Fas
 STD Fiber HFHP HFHP to Fiber
 STD Fiber HFHP HFHP to Fiber
 STD Fiber HFHP HFHP to Fiber
Exp 1: Actin
Exp 2: Actin
Exp 3: Actin
 STD Fiber HFHP HFHP to Fiber
 STD Fiber HFHP HFHP to Fiber
 STD Fiber HFHP HFHP to Fiber
Exp1: Claudin 5
Exp 3: Claudin 5
Exp 2: Claudin 5
 STD Fiber HFHP HFHP to Fiber
 STD Fiber HFHP HFHP to Fiber
 STD Fiber HFHP HFHP to Fiber
