# Supplementary material for: Diet effects on colonic health influence the efficacy of Bin1 mAb immunotherapy for ulcerative colitis
Source: Sci Rep. 2023 Jul 21;13:11802. doi: 10.1038/s41598-023-38830-2 (PMC10361997; doi:10.1038/s41598-023-38830-2)
Supplement: Supplementary file 3 — Supplementary Table S1. [file 41598_2023_38830_MOESM3_ESM.docx]

**Table 1. Standard Diet**

| **Ingredients** | **Nutritional Profile** | |
| --- | --- | --- |
| Whole wheat | ***Protein %: 19*** | ***Fat %: 12*** |
| Dehulled soybean meal | Arginine:1.15 | Linoleic acid:1.96 |
| Ground corn | Histidine: 0.46 | Linolenic acid: 0.15 |
| Wheat germ | Isoleucine: 0.83 | Arachidonic acid: 0.03 |
| Brewers dried yeast | Leucine: 1.4 | Omega-3 fatty acids:0.21 |
| Porcine animal fat | Lysine: 1.05 | Total saturated fatty acid: 3.72 |
| Condensed whey | Methionine: 0.62 | Total monounsaturated fatty acids: 3.96 |
| Soybean oil | Cystine: 0.36 | Polyunsaturated fatty acids: 0.64 |
| Dried whey protein concentrate | Phenylalanine: 0.84 | Cholesterol, ppm: 32 |
| (The percentage of the ingredients used in the diet is proprietary) | Tyrosine: 0.53 |  |
|  | Threonine: 0.71 | ***Minerals %*** |
|  | Tryptophan: 0.24 | Calcium: 0.80 |
|  | Valine: 0.88 | Phosphorus: 0.5 |
|  | Alanine: 0.29 | Potassium: 0.83 |
|  | Aspartic acid: 2.06 | Magnesium: 0.16 |
|  | Glutamic acid: 4.2 | Sodium: 0.43 |
|  | Glycine: 0.80 | Chloride: 0.70 |
|  | Proline: 1.3 | Sulfur: 0.27 |
|  | Serine: 1.01 | Fluorine, ppm:6.5 |
|  | Taurine: 0.0 | Iron, ppm: 160 |
|  |  | Zinc, ppm: 120 |
|  | ***Vitamins*** | Manganese, ppm: 120 |
|  | Vitamin A, IU/g: 18 | Copper, ppm: 18 |
|  | Vitamin D, IU/g: 3.3 | Cobalt, ppm: 0.63 |
|  | Vitamin E, IU/Kg: 66 | Iodine, ppm: 1.4 |
|  | Vitamin K, ppm: 3 | Chromium, ppm: 0.02 |
|  | Thiamin, ppm: 13 | Selenium, ppm: 0.30 |
|  | Riboflavin, ppm: 5.6 |  |
|  | Niacin, ppm: 74 | **Fiber %: 2.4** |
|  | Pantothenic acid, ppm: 20 |  |
|  | Folic acid, ppm: 2.9 | **Carbohydrates %: 52** |
|  | Pyridoxine, ppm: 9.6 |  |
|  | Biotin, ppm: 0.3 | **Energy (kcal/g): 3.60** |
|  | Vitamin B12, mcg/Kg: 51 | Protein: 19.75% |
|  | Choline chloride, ppm: 2000 | Fat:26.1% |
|  | Ascorbic acid, ppm: 0 | Carbohydrate: 54.15 |
|  |  |  |
