# Supplementary material for: Diet effects on colonic health influence the efficacy of Bin1 mAb immunotherapy for ulcerative colitis
Source: Sci Rep. 2023 Jul 21;13:11802. doi: 10.1038/s41598-023-38830-2 (PMC10361997; doi:10.1038/s41598-023-38830-2)
Supplement: Supplementary file 4 — Supplementary Table S2. [file 41598_2023_38830_MOESM4_ESM.docx]

**Table 2. Fiber Diet**

| **Ingredients %** | **Nutritional Profile** | |
| --- | --- | --- |
| Corn starch: 37.09 | ***Protein %: 10*** | ***Fat %: 4.1*** |
| Maltodextrin: 13.2 | Arginine: 0.37 | Linoleic acid:1.21 |
| Pectin: 12 | Histidine: 0.27 | Linolenic acid: 0.16 |
| Casein: 10.65 | Isoleucine: 0.51 | Arachidonic acid: 0.0 |
| Sucrose: 10 | Leucine: 0.92 | Omega-3-fatty acids:0.16 |
| Inulin: 4 | Lysine: 0.77 | Total saturated fatty acid: 1.13 |
| Powdered cellulose: 4 | Methionine: 0.27 | Total monounsaturated fatty acids: 1.28 |
| Mineral mix: 3.5 | Cystine: 0.34 | Polyunsaturated fatty acids: 1.28 |
| Soybean oil: 2 | Phenylalanine: 0.51 | Cholesterol, ppm: 19 |
| Lard: 2 | Tyrosine: 0.54 |  |
| Vitamin mix: 1 | Threonine: 0.41 | ***Minerals %*** |
| L-Cystine: 0.3 | Tryptophan: 0.12 | Calcium: 0.51 |
| Choline Bitartrate: 0.25 | Valine: 0.61 | Phosphorus: 0.24 |
| t-Butylhydroquinone: 0.0014 | Alanine: 0.29 | Potassium: 0.36 |
|  | Aspartic acid: 0.69 | Magnesium: 0.05 |
|  | Glutamic acid: 2.18 | Sodium: 0.13 |
|  | Glycine: 0.21 | Chloride: 0.22 |
|  | Proline: 1.26 | Fluorine, ppm:1.0 |
|  | Serine: 0.59 | Iron, ppm: 39 |
|  | Taurine: 0.0 | Zinc, ppm: 32 |
|  |  | Manganese, ppm: 11 |
|  | ***Vitamins*** | Copper, ppm: 6 |
|  | Vitamin A, IU/g: 4 | Cobalt, ppm: 0 |
|  | Vitamin D-3, IU/g: 1 | Iodine, ppm: 0.21 |
|  | Vitamin E, IU/Kg: 76.9 | Chromium, ppm: 1.0 |
|  | Vitamin K, ppm: 0.75 | Molybdenum, ppm: 0.14 |
|  | Thiamin, ppm: 4.8 | Selenium, ppm: 0.21 |
|  | Riboflavin, ppm: 6.4 |  |
|  | Niacin, ppm: 30 | **Fiber %: 19.6** |
|  | Pantothenic acid, ppm: 15 |  |
|  | Folic acid, ppm: 2.1 | **Carbohydrates %: 64.4** |
|  | Pyridoxine, ppm: 5.8 |  |
|  | Biotin, ppm: 0.2 | **Energy (kcal/g): 3.34** |
|  | Vitamin B12, mcg/Kg: 26 | Protein (kcal: 0.4): 12% |
|  | Choline chloride, ppm: 1250 | Fat (kcal: 0.367): 11% |
|  | Ascorbic acid, ppm: 0 | Carbohydrate (kcal:2.6): 77.1 |
|  |  |  |
