# Supplementary material for: Diet effects on colonic health influence the efficacy of Bin1 mAb immunotherapy for ulcerative colitis
Source: Sci Rep. 2023 Jul 21;13:11802. doi: 10.1038/s41598-023-38830-2 (PMC10361997; doi:10.1038/s41598-023-38830-2)
Supplement: Supplementary file 5 — Supplementary Table S3. [file 41598_2023_38830_MOESM5_ESM.docx]

**Table 3. Carbohydrate Diet**

| **Ingredients %** | **Nutritional Profile** | |
| --- | --- | --- |
| Corn starch: 48.8 | ***Protein %: 10*** | ***Fat %: 2.1*** |
| Maltodextrin: 16.2 | Arginine: 0.37 | Linoleic acid:0.61 |
| Sucrose: 12.2 | Histidine: 0.27 | Linolenic acid: 0.08 |
| Casein: 10.5 | Isoleucine: 0.50 | Arachidonic acid: 0.0 |
| Mineral mix: 3.5 | Leucine: 0.91 | Omega-3-fatty acids:0.08 |
| Pectin: 3 | Lysine: 0.76 | Total saturated fatty acid: 0.57 |
| Powdered cellulose: 1 | Methionine: 0.27 | Total monounsaturated fatty acids: 0.64 |
| Inulin: 1 | Cystine: 0.34 | Polyunsaturated fatty acids: 0.64 |
| Soybean oil: 1 | Phenylalanine: 0.5 | Cholesterol, ppm: 10 |
| Lard: 1 | Tyrosine: 0.53 |  |
| Vitamin mix: 1 | Threonine: 0.41 | ***Minerals %*** |
| L-Cystine: 0.3 | Tryptophan: 0.12 | Calcium: 0.51 |
| Choline Bitartrate: 0.25 | Valine: 0.6 | Phosphorus: 0.24 |
| t-Butylhydroquinone: 0.0014 | Alanine: 0.29 | Potassium: 0.36 |
|  | Aspartic acid: 0.68 | Magnesium: 0.05 |
|  | Glutamic acid: 2.15 | Sodium: 0.13 |
|  | Glycine: 0.20 | Chloride: 0.22 |
|  | Proline: 1.24 | Fluorine, ppm:1.0 |
|  | Serine: 0.58 | Iron, ppm: 39 |
|  | Taurine: 0.0 | Zinc, ppm: 32 |
|  |  | Manganese, ppm: 11 |
|  | ***Vitamins*** | Copper, ppm: 6 |
|  | Vitamin A, IU/g: 4 | Cobalt, ppm: 0 |
|  | Vitamin D-3, IU/g: 1 | Iodine, ppm: 0.21 |
|  | Vitamin E, IU/Kg: 75.9 | Chromium, ppm: 1.0 |
|  | Vitamin K, ppm: 0.75 | Molybdenum,ppm: 0.14 |
|  | Thiamin, ppm: 4.8 | Selenium, ppm: 0.21 |
|  | Riboflavin, ppm: 6.4 |  |
|  | Niacin, ppm: 30 | **Fiber %: 4.9** |
|  | Pantothenic acid, ppm: 15 |  |
|  | Folic acid, ppm: 2.1 | **Carbohydrates %: 78.5** |
|  | Pyridoxine, ppm: 5.8 |  |
|  | Biotin, ppm: 0.2 | **Energy (kcal/g): 3.73** |
|  | Vitamin B12, mcg/Kg: 26 | Protein (kcal: 0.4): 10.7% |
|  | Choline chloride, ppm: 1250 | Fat (kcal: 0.189): 5.1% |
|  | Ascorbic acid, ppm: 0 | Carbohydrate (kcal:3.14): 84.2 |
|  |  |  |
