# Supplementary material for: Diet effects on colonic health influence the efficacy of Bin1 mAb immunotherapy for ulcerative colitis
Source: Sci Rep. 2023 Jul 21;13:11802. doi: 10.1038/s41598-023-38830-2 (PMC10361997; doi:10.1038/s41598-023-38830-2)
Supplement: Supplementary file 6 — Supplementary Table S4. [file 41598_2023_38830_MOESM6_ESM.docx]

**Table 4. High Fat / High Protein Diet**

| **Ingredients %** | **Nutritional Profile** | |
| --- | --- | --- |
| Casein: 33.7 | ***Protein %: 30*** | ***Fat %: 28.5*** |
| Lard: 25.5 | Arginine: 1.18 | Linoleic acid:4 |
| Maltodextrin: 16.2 | Histidine: 0.87 | Linolenic acid: 0.34 |
| Sucrose: 12.32 | Isoleucine: 1.61 | Arachidonic acid: 0.05 |
| Corn starch: 4.18 | Leucine: 2.91 | Omega-3-fatty acids:0.34 |
| Mineral mix: 3.5 | Lysine: 0.77 | Total saturated fatty acid: 11.08 |
| Soybean oil: 3 | Methionine: 0.27 | Total monounsaturated fatty acids: 11.39 |
| Vitamin mix: 1 | Cystine: 0.34 | Polyunsaturated fatty acids: 4.23 |
| L-Cystine: 0.3 | Phenylalanine: 0.51 | Cholesterol, ppm: 242 |
| Choline Bitartrate: 0.25 | Tyrosine: 0.54 |  |
| t-Butylhydroquinone: 0.0014 | Threonine: 0.41 | ***Minerals %*** |
|  | Tryptophan: 0.12 | Calcium: 0.51 |
|  | Valine: 0.61 | Phosphorus: 0.43 |
|  | Alanine: 0.29 | Potassium: 0.36 |
|  | Aspartic acid: 0.69 | Magnesium: 0.05 |
|  | Glutamic acid: 2.18 | Sodium: 0.12 |
|  | Glycine: 0.21 | Chloride: 0.21 |
|  | Proline: 1.26 | Fluorine, ppm:1.0 |
|  | Serine: 0.59 | Iron, ppm: 39 |
|  | Taurine: 0.0 | Zinc, ppm: 39 |
|  |  | Manganese, ppm: 11 |
|  | ***Vitamins*** | Copper, ppm: 6 |
|  | Vitamin A, IU/g: 4 | Cobalt, ppm: 0 |
|  | Vitamin D-3, IU/g: 1 | Iodine, ppm: 0.21 |
|  | Vitamin E, IU/Kg: 77.8 | Chromium, ppm: 1.0 |
|  | Vitamin K, ppm: 0.75 | Molybdenum,ppm: 0.14 |
|  | Thiamin, ppm: 4.8 | Selenium, ppm: 0.28 |
|  | Riboflavin, ppm: 7.2 |  |
|  | Niacin, ppm: 30 | **Fiber %: 0** |
|  | Pantothenic acid, ppm: 17 |  |
|  | Folic acid, ppm: 2.2 | **Carbohydrates %: 32.9** |
|  | Pyridoxine, ppm: 5.8 |  |
|  | Biotin, ppm: 0.2 | **Energy (kcal/g): 5.08** |
|  | Vitamin B12, mcg/Kg: 30 | Protein (kcal: 1.2): 23.6% |
|  | Choline chloride, ppm: 1250 | Fat (kcal: 2.56): 50.5% |
|  | Ascorbic acid, ppm: 0 | Carbohydrate (kcal:1.31): 25.9 |
|  |  |  |
